# Screening of linear B-cell epitopes and its proinflammatory activities of *Haemophilus parasuis* outer membrane protein P2

**DOI:** 10.3389/fcimb.2023.1192651

**Published:** 2023-05-03

**Authors:** Jingbo Wu, Wenjin Nan, Guoliang Peng, Honghui Hu, Chongbo Xu, Jianqiang Huang, Zhengzhong Xiao

**Affiliations:** ^1^ Henry Fok College of Yingdong Biology and Agricultural, Shaoguan University, Shaoguan, China; ^2^ North Guangdong Collaborative Innovation and Development Center of Pig Farming and Disease Control, Shaoguan University, Shaoguan, China; ^3^ North Guangdong Pig Breeding Waste Reduction Engineering Technology Center, Shaoguan University, Shaoguan, China

**Keywords:** *Haemophilus parasuis*, linear B-cell epitopes, OmpP2 protein, proinflammatory cytokines, genotype

## Abstract

*Haemophilus parasuis* is a commensal organism of the upper respiratory tract of pigs, but virulent strains can cause Glässer’s disease, resulting in significant economic losses to the swine industry. OmpP2 is an outer membrane protein of this organism that shows considerable heterogeneity between virulent and non-virulent strains, with classification into genotypes I and II. It also acts as a dominant antigen and is involved in the inflammatory response. In this study, 32 monoclonal antibodies (mAbs) against recombinant OmpP2 (rOmpP2) of different genotypes were tested for reactivity to a panel of OmpP2 peptides. Nine linear B cell epitopes were screened, including five common genotype epitopes (Pt1a, Pt7/Pt7a, Pt9a, Pt17, and Pt19/Pt19a) and two groups of genotype-specific epitopes (Pt5 and Pt5-II, Pt11/Pt11a, and Pt11a-II). In addition, we used positive sera from mice and pigs to screen for five linear B-cell epitopes (Pt4, Pt14, Pt15, Pt21, and Pt22). After porcine alveolar macrophages (PAMs) were stimulated with overlapping OmpP2 peptides, we found that the epitope peptides Pt1 and Pt9, and the loop peptide Pt20 which was adjacent epitopes could all significantly upregulated the mRNA expression levels of *IL-1α, IL-1β, IL-6, IL-8*, and *TNF-α*. Additionally, we identified epitope peptides Pt7, Pt11/Pt11a, Pt17, Pt19, and Pt21 and loop peptides Pt13 and Pt18 which adjacent epitopes could also upregulate the mRNA expression levels of most proinflammatory cytokines. This suggested that these peptides may be the virulence-related sites of the OmpP2 protein, with proinflammatory activity. Further study revealed differences in the mRNA expression levels of proinflammatory cytokines, including *IL-1β* and *IL-6*, between genotype-specific epitopes, which may be responsible for pathogenic differences between different genotype strains. Here, we profiled a linear B-cell epitope map of the OmpP2 protein and preliminarily analyzed the proinflammatory activities and effects of these epitopes on bacterial virulence, providing a reliable theoretical basis for establishing a method to distinguish strain pathogenicity and to screen candidate peptides for subunit vaccines.

## 1 Introduction

*Haemophilus parasuis* (*H. parasuis*) is a normal inhabitant of the upper respiratory tract of pigs. Some virulent strains of this organism break through the mucosal barrier and invade the body, causing Glässer disease, which is characterized by pneumonia, fibrous polyserous disease, polyarthritis, meningitis, and other inflammatory conditions (Oliveira and Pijoan, 2004). *H. parasuis* is the main pathogen causing respiratory diseases in swine, accounting for 28.54% of all porcine respiratory diseases in China (Sun et al., 2022). *H. parasuis* seriously threatens nursery pigs, with an incidence of 10%–15% and a mortality rate of 50% (Zimmerman et al., 2012). Morbidity and mortality further increase in cases of mixed viral infections (Costa-Hurtado and Aragon, 2013). Therefore, *H. parasuis* virulent strain infections have emerged as a major cause of death in nursery pigs, causing enormous economic losses in the swine industry worldwide(Costa-Hurtado and Aragon, 2013).

*H. parasuis* is generally divided into 15 serotypes, and serovars are commonly used as indicators of virulence (Kielstein and Rapp-Gabrielson, 1992). However, the virulence manifestations caused by different strains of the same serovar, or in different pigs challenged by the same strain, vary (Brockmeier et al., 2013; Brockmeier et al., 2014; Schuwerk et al., 2020). This suggests that there are other virulence markers in addition to serotypes in *H. parasuis* (Schuwerk et al., 2020). An outer membrane protein in the range of 36.6–38.5 kDa may be related to virulence, based on comparisons of the *H. parasuis* strains isolated from healthy and diseased pigs. The length of this outer membrane protein P2 (OmpP2) is 359–401 amino acids (aa) and its molecular weight is consistent with this range (Ruiz et al., 2001; Mullins et al., 2009). OmpP2 is the most abundant outer membrane protein in *H. parasuis* and belongs to the porin family, which is the main structural protein and a nutrient circulation channel in bacteria (Zhou et al., 2009b; Costa-Hurtado and Aragon, 2013; Zhang et al., 2014). *H. parasuis* virulent strains with OmpP2-deficient mutants not only have pronounced growth defects (Zhang et al., 2012a), but also have significantly lower adherence capacity and serum resistance than the parent strains (Zhang et al., 2012a; Zhang et al., 2012b). In addition, OmpP2 is also cytotoxic (Zhao et al., 2010), and can induce cells to secrete proinflammatory cytokines, such as IL-1α, IL-1β, IL-6, and IL-8 (Zhou et al., 2014). These results demonstrate that OmpP2 is one of the main virulence factors of *H. parasuis*, and is involved in most processes of spread and pathogenicity of *H. parasuis* in the host.

OmpP2 has two distinct protein structures and its corresponding genes are divided into two genotypes (I and II) (Mullins et al., 2009; Zhao et al., 2010; Li et al., 2012). According to the predicted protein structure, there are two discontinuous gene deletions in the genotype I protein, which results in the absence of loop9 between loop3 and loop4 in the tertiary structure of the genotype I protein, and with a shift in loop5 by 14 aa toward the C-terminus (Mullins et al., 2009). Combined analysis of the genotype and virulence of the strains showed that the OmpP2 of most medium-virulence strains, strong-virulence strains, and clinically isolated strains belonged to genotype I, while weak-virulence strains and non-virulent strains belonged to genotype II (Wu et al., 2016). Animal and cytotoxicity experiments have also shown that genotype I OmpP2 has higher cytotoxicity and serum resistance (Cerda-Cuellar and Aragon, 2008; Zhao et al., 2010), indicating that the structure of the OmpP2 protein is strongly associated with, and can be used as a reliable basis for distinguishing strain virulence.

The exact mechanism by which changes in the OmpP2 structure affect strain virulence remains unclear. It has been confirmed that mutations in the amino acid sequence of the surface exposure loop or antigenic determinants of porin proteins can lead to changes in the pathogenicity of some Gram-negative bacteria (Galdiero et al., 2012). For example, *H. influenzae*, which belongs to the same genus as *H. parasuis*, also contains an OmpP2 protein with similar structure and function. It has previously been established that loop7 of *H. influenzae* OmpP2 can increase *IL-6* and *TNF-α* mRNA expression levels and participates in the inflammatory response; however, a mutated loop7 lack this ability (Galdiero et al., 2006). A recent study showed that loop7 and loop8 of OmpP2 from the *H. parasuis* wild-type strain can also upregulate mRNA levels of *IL-1α, IL-1β, IL-6, IL-8, IL-17, IL-23, CCL4*, and *CCL5* in alveolar macrophages, suggesting that they form the active region of OmpP2, with proinflammatory activities. However, it remains unknown whether differences in the loops from different OmpP2 genotypes have an impact on proinflammatory activity (Zhou et al., 2019). In addition, loop5 of OmpP2 of *H. influenzae* is the binding site of most functional antibodies (Yi and Murphy, 1997), while loop6 is the main binding site of bactericidal antibodies (Neary et al., 2001). Both of these have B-cell epitopes. *H. influenzae* can survive and evade immune clearance in the lungs with the help of loop5 and loop6 mutations, causing chronic obstructive pulmonary disease, and demonstrating that mutations in exposed epitopes can also alter the virulence of *H. influenzae* (Regelink et al., 1999). It has been determined that OmpP2 of *H. parasuis* is also a dominant antigen that can rapidly induce the production of high-titer, specific antibodies, effectively kill bacteria in the presence of complement factors, and inhibit the growth of bacteria in the host (Zhou et al., 2009a), suggesting that the B cell epitope of OmpP2 may also be correlated with pathogenicity. However, the exact epitope map and its impact on pathogenicity remain poorly understood.

In this study, we prepared mAbs against OmpP2 of different genotypes, which were used to screen for genotype-common (GC) and genotype-specific (GS) epitopes. In addition, we used overlapping peptides covering the full-length sequence of OmpP2 to stimulate PAMs and investigated the roles of the epitopes and loops of *H. parasuis* OmpP2 in the inflammatory response.

## 2 Materials and methods

### 2.1 Bacteria, cells, and culture conditions

*Escherichia coli* DH5α and BL21(DE3) were purchased from Takara Bio Inc (Beijing, China) and grown in Luria–Bertani medium. *H. parasuis* DT3 and ZS7 strains (Laboratory collection) were isolated from the lung tissue and nasal cavity of different diseased pigs in southern China, respectively; and were grown at 37°C in an aeroculture in tryptic soy broth and tryptic soy agar, supplemented with 50 μg/mL nicotinamide adenine dinucleotide (NAD) and 5% inactivated equine serum.

PAMs (3D4/21, ATCC, Manassas, VA, USA) and SP2/0 myeloma cells were purchased from Otwo Biotech Inc. (Guangdong, China). PAMs were cultured in Dulbecco’s Modified Eagle’s medium (DMEM) containing 10% fetal bovine serum (FBS) and 1 × penicillin–streptomycin solution (PS). SP2/0 cells were grown in RPMI 1640 medium supplemented with 10% FBS and 1 × PS. All cells were grown at 37°C in a 5% CO_2_ atmosphere. All the cell culture media and supplements were purchased from Thermo Fisher Scientific (Waltham, MA, USA). The cells were subcultured until they reached an 80%–90% confluence.

### 2.2 Preparation of rOmpP2 and native OmpP2

Recombinant truncated OmpP2 (rOmpP2) without the signal peptide was expressed using the prokaryotic expression vector pET-28a. Different rOmpP2 genes were amplified from purified genomic DNA of clinical samples (nasal swabs, lungs, lymph nodes, joint effusion, etc.) from healthy or diseased pigs, with the primer set 5′-CATGCCATGGTAACAGTTTATGAAAATGAAGGT-3′ and 5′-CCGCTCGAGCCATAATACACGTAAACC-3′, to ensure that a 6 × His-tag was attached to the C-terminal. The expressed fusion proteins were located within inclusion bodies. The inclusion bodies were washed, the protein solubilized, and then refolded by dialysis against a decreasing concentration gradient (6 M, 4 M, 2 M, 0 M) of urea at 4°C. The recombinant proteins were separated by 10% sodium dodecyl sulfate–polyacrylamide gel electrophoresis (SDS-PAGE) after concentration by ultrafiltration (Millipore, Billerica, MA, USA). They were identified by Coomassie bright-blue staining or western blotting (WB) using an anti-histidine tag antibody (Sangon Biotech, Shanghai, China). Protein concentration was determined using the BCA Protein Assay Kit (Sangon Biotech), and protein purity was automatically calculated by the image tools of GelDoc XR+ IMAGELAB (BioRad, Hercules, CA, USA). rOmpP2 proteins were used as antigens for mouse immunization and serological detection.

Native OmpP2 (nOmpP2) proteins were extracted from the *H. parasuis* DT3 and ZS7 strains as previously described (Zhang et al., 2012b; Zhou et al., 2014), and were confirmed to be genotype I by DNA sequencing.

### 2.3 Peptides

To locate the B cell epitopes of different genotypes of OmpP2, three groups of overlapping peptides (**Table 1**) were designed and synthesized by Sangon Biotech. In the first group, twenty-one 21–24-mer peptides with 8-mer overlaps were designated as Pt1 to Pt21, based on the full-length gene of genotype I rOmpP2 (the synthesis of Pt10 failed). In addition, three peptides (Pt22, Pt23, and Pt24) were designed based on the insertion sequence of genotype II rOmpP2. In the second group, eight peptides were designed based on the predicted linear B-cell epitopes and loops (epitope prediction: IEDB software, http://tools.iedb.org/bcell/; loop prediction: PRED-TMBB software, http://bioinformatics.biol.uoa.gr/PRED-TMBB/input.jsp) of the genotype I rOmpP2 protein, to complement the first group and to cover all the predicted epitopes and loops. In the third group, based on the peptide–enzyme-linked immunosorbent assay (ELISA) results from the first and second groups, nine peptides were designed to confirm the position or genotype-specificity of the epitope. All peptides were 90% pure and were dissolved in sterile water (poorly soluble peptides were completely dissolved by ultrasound treatment then filtered through 0.22 μm filters).

**Table 1:** Sequence of overlapping peptides of OmpP2 protein.

Peptide	Sequence	Length	Position
Group A			
Pt1	VTVYENEGTKVDFDGQLRLLLEKQ	24	rP2-I2(1-24)
Pt2	LRLLLEKQASKVKGQSSTSGHTDL	24	rP2-I2(17-40)
Pt3	STSGHTDLKNNGSRFGISIKHNIN	24	rP2-I2(33-56)
Pt4	ISIKHNINENLYGFGRYETRLGRN	24	rP2-I2(49-72)
Pt5	YETRLGRNSKNDAGWGDVTTDEAY	24	rP2-I2(65-88)
Pt6	DVTTDEAYVGLGGYGHEISFGKQA	24	rP2-I2(81-104)
Pt7	EISFGKQAVIGDSIGQAGFDKVYG	24	rP2-I2(97-120)
Pt8	AGFDKVYGVGTGGIKYSANNTNKK	24	rP2-I2(113-136)
Pt9	SANNTNKKGFDILTASSDSAINYT	24	rP2-I2(129-152)
Pt10	SDSAINYTYTGIEGLTLGANYNVA	24	rP2-I2(145-168)
Pt11	LGANYNVANERDNKGEVKVDSTKS	24	rP2-I2(161-184)
Pt12	VKVDSTKSGFGLGAKYTAKIAESQ	24	rP2-I2(177-200)
Pt13	TAKIAESQSVTVAAGYTHDDYKSG	24	rP2-I2(193-216)
Pt14	THDDYKSGSVNKKDKDGVYFGLKY	24	rP2-I2(209-232)
Pt15	GVYFGLKYVNAPFTVAVDGGHGVE	24	rP2-I2(225-248)
Pt16	VDGGHGVEKTGNVKEKIDFVRTGA	24	rP2-I2(241-264)
Pt17	IDFVRTGARFDVTPKSGVYGNYSY	24	rP2-I2(257-280)
Pt18	GVYGNYSYGTYKNKAYKATAHQFM	24	rP2-I2(273-296)
Pt19	KATAHQFMLGADYKLHKQVVTFVE	24	rP2-I2(289-312)
Pt20	KQVVTFVEGRLIKNKDSNNNKVTD	24	rP2-I2(305-328)
Pt21	SNNNKVTDKALGVGLRVLWLE	21	rP2-I2(321-341)
Pt22	TYKVDESITVNNTQGTFKYSAPQE	24	rP2-II(129-152)
Pt23	GTFKYSAPQEGFDILTQSSDSA	22	rP2-II(143-164)
Pt24	QLKGKFVQANGTSTDHTYTESF	22	rP2-II(234-255)
Group B			
Pt1a	VTVYENEGTKVDFDGQ	16	rP2-I2(1-16)
Pt2a	LEKQASKVKGQSSTSGHTDLKNNGSR	26	rP2-I2(21-46)
Pt3a	KNNGSRFGISIKHNINENLYGFGR	24	rP2-I2(41-64)
Pt7a	GKQAVIGDSIGQAGFDKVYGVGTGGIKY	28	rP2-I2(101-128)
Pt11a	NYNVANERDNKGEVKVDSTKSGFGLGA	27	rP2-I2(164-190)
Pt12a	GLGAKYTAKIAESQSVTVAAGY	22	rP2-I2(187-208)
Pt19a	YKLHKQVVTFVEGR	14	rP2-I2(301-314)
Pt20a	LIKNKDSNNNKVTDKALGVGL	21	rP2-I2(315-335)
Group C			
Pt5a	RLGRNSKNDAGW	12	rP2-I2(68-79)
Pt5b	AGWGDVTT	8	rP2-I2(77-84)
Pt5-I3	YETRLDSNSENAAGWGDVKTKYAY	24	rP2-I3(65-88)
Pt5-II	YETRLGSGSKNAAKWGDVTTDEA	23	rP2-II(65-88)
Pt9a	GFDILTASSDSAINYT	16	rP2-I2(137-152)
Pt9b	SSDSAINYT	9	rP2-I2(144-152)
Pt11b	NERDNKGEV	9	rP2-I2(169-177)
Pt11c	VKVDSTKS	8	rP2-I2(177-184)
Pt11a-II	NYNVANEREKADVKVDSIKSGFGLGA	26	rP2-II(180-205)

### 2.4 Preparation of mAbs against OmpP2

To obtain mAbs against different genotypes of the OmpP2 protein, two mouse immunization groups were set up and immunized with rP2-I2 and rP2-II. Three 6-week-old female BALB/c mice were used in each group. The first subcutaneous injection contained 100 μg rOmpP2 protein emulsified in equal proportions with Freund’s complete adjuvant (Sigma–Aldrich, St Louis, MO, United States). Two weeks later, the second subcutaneous injection, containing 50 μg rOmpP2 protein emulsified in equal proportions with Freund’s incomplete adjuvant (Sigma–Aldrich) was administered. Immunizations were performed two or more times at 2-week intervals, depending on the antibody titer, which was assessed by indirect ELISA using rOmpP2 as the antigen. If the antibody titer exceeded 12,800, the final boost immunization, of 50 μg of rOmpP2 in phosphate-buffered saline (PBS), was intraperitoneally injected.

Three days after the final immunization, spleen cells were aseptically harvested and fused with SP2/0 cells at a ratio of 3:1 using polyethylene glycol 1450. Supernatants from the antibody-producing hybridomas were screened by indirect ELISA using wells coated with the antigens injected into each immunization group. Positive hybridomas were selected and subcloned three times using limited dilution. All the clones from the same fusion cell well were treated as positive hybridoma cell lines.

### 2.5 Indirect ELISA

To screen for positive hybridoma cell clones and linear B cell epitopes, indirect ELISA was performed as previously described (Qiu et al., 2012), with some modifications. Briefly, immuno-microtiter plates were coated with 5 μg/mL of peptides or 3 μg/mL of proteins in carbonate-buffered saline (pH = 9.6) at 4°C overnight. Plates were washed three times with PBST (0.01 M PBS, pH 7.2; 0.05% Tween 20) and blocked with 4% bovine serum albumin at 37°C for 2 h. Hybridoma cell supernatant (1:1) and serum (1:50 to 1:102,400) were diluted and added to the plate in a volume of 100 μL/well and incubated at 37°C for 1 h. After washing five times with PBST, horseradish peroxidase (HRP)-conjugated rabbit anti-mouse IgG (1:10,000 diluted) was added as the secondary antibody and plates were incubated at 37°C for 45 min. After washing five times with PBST, 100 μL chromogenic solution (TMB, Sangon Biotech) was added and incubated at 37°C for 20 min. The absorbance was measured using a microplate reader at an absorption of 450 nm. An mAb to the P30 protein of African swine fever virus (ASFV) was used as a negative control, and test results were calculated as the sample to cut-off (S/CO) ratio: S/CO value = sample detection value/cutoff (CO) value, where the CO value = 2.1 × negative-control detection value. An S/CO ratio ≥ 1.0 was considered as positive.

### 2.6 Western blotting

rOmpP2 and nOmpP2 were separated by SDS-PAGE and transferred to polyvinylidene fluoride membranes. After blocking overnight at 4°C in 5% skim milk, the protein-bound membranes were incubated with the diluted supernatants of mAbs for 1 h at room temperature. After washing, the membranes were incubated with HRP-conjugated rabbit anti-mouse IgG (1:10000 diluted) for 1 h at room temperature. Finally, the proteins were detected by adding an enhanced chemiluminescence reagent (Thermo Fisher Scientific). The P30 protein of ASFV was used as a negative control.

### 2.7 Epitopes sequence alignment and characterization

The nOmpP2 genes of the DS3 and ZS7 strains and the rOmpP2 genes were amplified and sequenced using the primers described above. Thirty OmpP2 protein sequences of 15 serovars from *H. parasuis* reference strains (15 international reference strains and 15 Chinese reference strains) were obtained from Genbank (Additional file 2). Sequence alignment was performed using MEGA 5.1 software, and the sequence consistency, mutation sites of each epitope locus, and the relationships among the epitope, genotype, and pathogenicity were analyzed. If an epitope existed only in a single genotype, it was defined as a GS epitope, and the mAb that interacted with it was defined as the GS-antibody (GS-Ab). If the epitope was present in both genotypes, it was defined as the GC epitope, and the mAb that interacted with it was defined as the genotype-common antibody (GC-Ab).

### 2.8 Clinical sample detection

A total of 187 swine serum samples were collected from nine farms and epitope peptide-ELISA was performed to analyze the clinical positivity rate of antibodies to each epitope. The ELISA protocol was similar to that previously described. Swine serum (diluted 1:400) was used as the primary antibody, and HRP-conjugated rabbit anti-pig IgG (Sangon Biotech; diluted 1:10,000) was used as the secondary antibody. The incubation time with the secondary antibody was reduced to 30 min, the incubation time with TMB was reduced to 10 min, and the other procedures remained unchanged. A mock control, without an antigen coating, was set up for each serum sample, and serum from newborn swine born to negative sows was used as the negative control. The S/CO value was calculated as follows: sample OD_450_ value (S) = OD_450_ for serum with peptides - OD_450_ for serum with mock; CO value = 2.1 × (OD_450_ for negative control with peptides - OD_450_ for negative control with mock). An S/CO ratio ≥ 1.0 was considered as positive. The 187 clinical serum samples were detected in parallel using a commercial *H. parasuis* antibody ELISA kit (Shenzhen Ziki Biotech Co., Shenzhen, China), which used a one-step assay with the serum diluted 1:15. We then compared the results of the commercial kit with those of the epitope peptide-ELISA to analyze the potential of the epitopes in clinical diagnosis. Finally, 20 double-positive serum samples (positive by commercial ELISA and epitope peptide-ELISA) were randomly selected and used as primary antibodies to perform an indirect ELISA with the remaining peptides to screen for missed epitopes in the mouse experiments by using polyclonal antibodies in pig serum.

### 2.9 PAM stimulation with OmpP2 peptides

Log-phase growing PAMs were seeded in 24-well plates at a density of 2 × 10^5^ cells/well and cultured overnight. When the cultures reached confluence, the cells were stimulated in triplicate with 130 nmol/mL peptides (diluted with serum-free DMEM) for 12 h. Untreated cells (cultured in serum-free DMEM) were used as mock controls in each assay. Following treatment, the cells were lysed and total RNA purified using the TRIzol method. cDNA was synthesized using the Evo M-MLV RT Kit with clean gDNA (Accurate Biology, Hunan, China). Real-time polymerase chain reaction (PCR) was performed to determine the relative quantities of *IL-1α, IL-1β, IL-6, IL-8*, *TNF-α*, and *GAPDH* mRNA, with SYBR Green Premix Pro Taq HS qPCR Kit (Accurate Biology) and ABI 7500 Real-Time PCR System (Applied Biosystems, CA, USA). The primers (**Table S1**) used for RT-PCR were synthesized by Sangon Biotech, Inc. Each sample was assessed in triplicate, in at least three independent experiments. Relative expression of the cytokine genes was normalized to the internal control gene (*GAPDH*), analyzed using the 2^-ΔΔCT^ method, and was presented as the fold-change relative to the mock control.

### 2.10 Statistical analysis

Statistical analysis of the data was conducted by one-way analysis of variance (ANOVA) using SPSS v16 software (IBM SPSS Inc., Armonk, NY, USA). The *post-hoc* Duncan test was used to identify differences between group means. *p* < 0.05 were considered significant.

## 3 Results

### 3.1 Expression and identification of immunogenic protein

**Figure 1:**
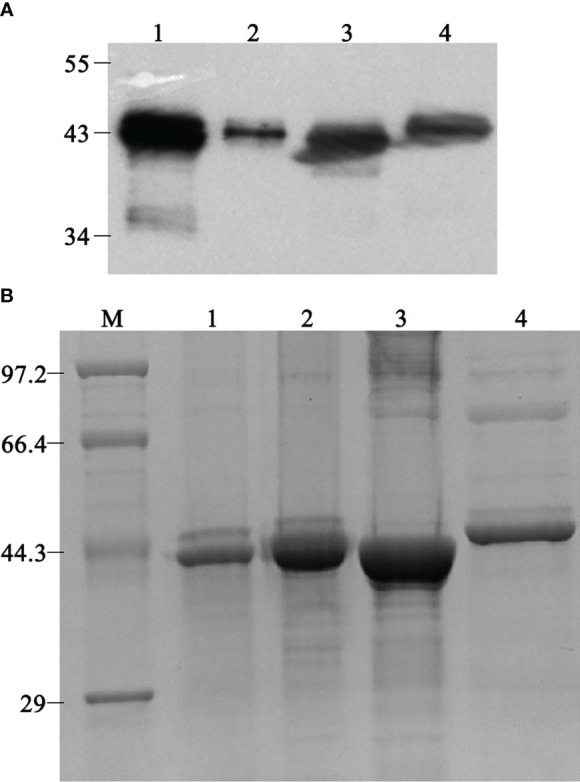
Western blotting and sodium dodecyl sulfate–polyacrylamide electrophoresis analysis of refolded rOmpP2. **(A)** The rOmpP2 proteins were detected with an anti-His tag antibody. **(B)** The rOmpP2 proteins were separated on 12% polyacrylamide gels and stained with Coomassie Brilliant Blue. Lanes 1–4 contain rP2-I1, rP2-I2, rP2-I3, and rP2-II, respectively; lane M, protein molecular mass marker (kDa).

Three different *ompP2* genes of genotype I were amplified from clinical samples of diseased pigs, and one *ompP2* gene of genotype II was amplified from a nasal swab of a healthy pig. An expression vector containing any one of the four *ompP2* genes was used to express heterologous proteins in *E. coli* BL21 (DE3) cells. Western blotting with an anti-His tag antibody confirmed that all four heterologous proteins contained a His tag (**Figure 1A**), indicating that the proteins were, as expected, rOmpP2 proteins. We named the three proteins of genotype I rP2-I1, rP2-I2, and rP2-I3 and the protein of genotype II as rP2-II. As expected, the apparent molecular weight of rOmpP2 proteins was 43–44.3 kDa, and the masses of the three genotype I proteins were similar and slightly smaller than that of the rP2-II protein. After being concentrated and verified by SDS-PAGE (**Figure 1B**), the concentrations of rP2-I1, rP2-I2, rP2-I3, and rP2-II were 2.9 mg/mL, 5.8 mg/mL, 5 mg/mL, and 1.9 mg/mL, respectively, with a purity of 90%, 94.6%, 93.2%, and 88.4% respectively. These results indicated that they recombinant proteins were adequate for use as immunogenic antigens. rP2-I2 (the highest and purest OmpP2 content among genotype I samples) and rP2-II were used for mouse immunization.

### 3.2 Production and identification of mAbs to OmpP2 protein

**Figure 2:**
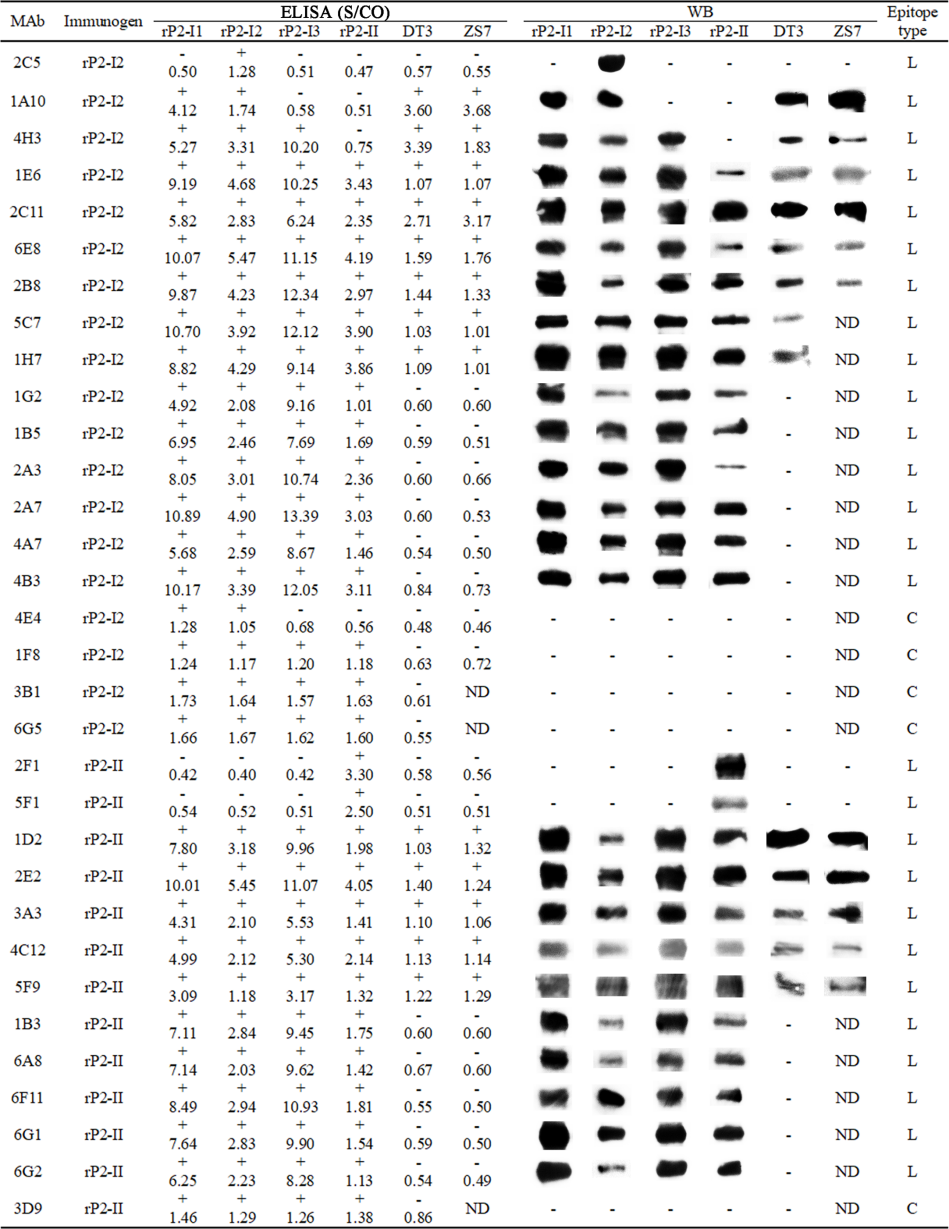
Enzyme-linked immunosorbent assay (ELISA) and western blotting (WB) analysis of monoclonal antibodies (mAbs). The number of the ELISA is the S/CO value: S/CO value ≥ 1 is positive (+) and ≤ 1 is negative (-); “ND” means “not done”, “L” means the antibody recognizes a linear epitope, and “C” means the antibody recognizes a conformational epitope.

After the second immunization, the antibody titers of mice immunized with rP2-I2 and rP2-II were 51,200 and 25,600, respectively. The mice were then sacrificed and their spleens were harvested for cell fusion. After three subcloning steps, 19 and 13 mAbs (32 in total) against OmpP2 were established by indirect ELISA and were selected from the rP2-I2 and rP2-II immunization groups, respectively. The reactivity and specificity of each mAb were confirmed by indirect ELISA and WB analysis with the four rOmpP2 proteins (**Figure 2**). Indirect ELISA revealed that 81.25% of the mAbs (rP2-I2, 15/19; rP2-II, 11/13) reacted with the four rOmpP2 proteins, showing that most of the mAbs could recognize genotype I and II OmpP2 proteins in the tertiary structure, which were defined as GC-Abs. Additionally, mAbs 2C5, 1A10, 4H3, and 4E4 from the rP2-I2 immunization group only recognized one or more proteins from genotype I, which were defined as GS-Abs of genotype I, accounting for 21.05% (4/19) of the mAbs from the rP2-I2 immunization group. In contrast, mAbs 2F1 and 5F1 from the rP2-II immunization group only recognized the rP2-II protein of genotype II, which was defined as a GS-Abs of genotype II, and accounted for 15.38% (2/13) of the mAbs from the rP2-II immunization group. The genotypes of the proteins recognized by the antibody used in the WB assay were consistent with the ELISA results. WB analysis indicated that 84.38% (27/32) of the mAbs could recognize the primary (linear) structure of the OmpP2 protein, suggesting that these mAbs reacted with linear B-cell epitopes. In addition, four mAbs (4E4, 1F8, 3B1, and 6G5) from the rP2-I2 immunization group and mAb 3D9 from the rP2-II immunization group recognized only the tertiary structure, accounting for 21.05% (4/19) and 7.69% (1/13) of the mAbs in the rP2-I2 and rP2-II immunization groups, respectively, indicating that these mAbs reacted with conformational B-cell epitopes.

### 3.3 B-cell linear epitope screening

**Figure 3:**
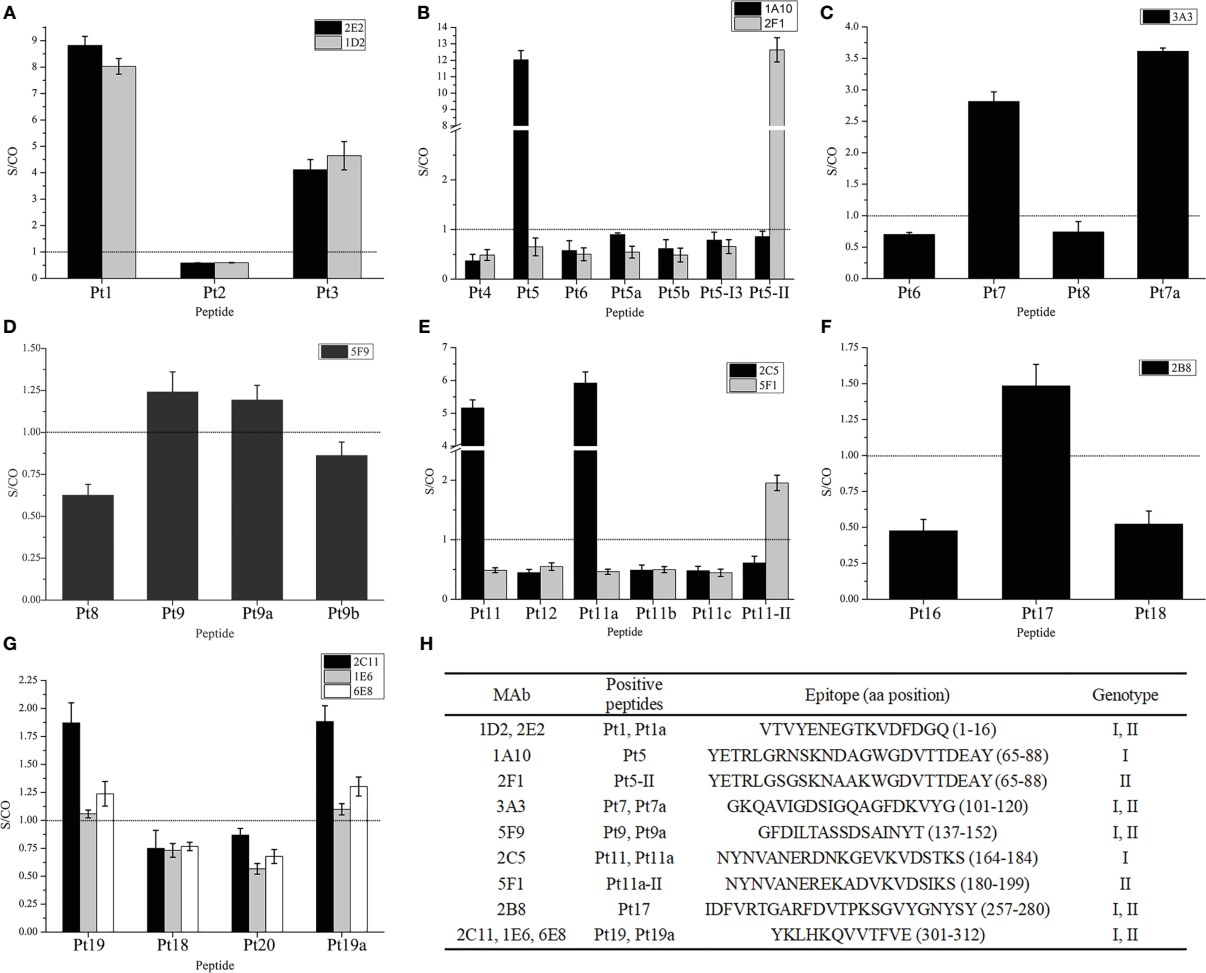
Reactivity of monoclonal antibodies to overlapping peptides. **(A–G)** Bindings of mAbs to overlapping peptides in the peptide–enzyme-linked immunosorbent assay (ELISA), the dotted line in the figure is the threshold line (S/CO = 1). **(H)** Information of the screening epitope. The amino acid position of peptides in parentheses were referenced to rP2-I2 protein sequences, except for Pt5-II and Pt11a-II, which are based on the sequence of rP2-II proteins. “Genotype” is the genotype of proteins to which peptides belong.

To confirm mAb recognition of the epitopes, 27 mAbs were detected for reactivity with 29 overlapping peptides in groups A and B. The results (**Figure 3**) indicated that 10 mAbs were positive for one or two peptides, including mAbs 1A10, 2C5, 1E6, 2C11, 6E8, and 2B8 from the rP2-I2 immunization group, and 2E2, 1D2, 3A3, and 5F9 from the rP2-II immunization group.

Among the GC-mAbs, mAbs 2E2 and 1D2 reacted with Pt1 and Pt1a, and the epitope recognized was the overlapping region Pt1a: VTVYENEGTKVDFDGQ (rP2-I2, 1–16 aa). mAb 3A3 reacted with Pt7 and Pt7a, and the recognized epitope was the overlapping region GKQAVIGDSIGQAGFDKVYG (rP2-I2, 101–120 aa). mAb 5F9 reacted with Pt9. We then further truncated Pt9 to Pt9a and Pt9b, and found that mAb 5F9 reacted with Pt9a but not with Pt9b; thus, the key region of epitope of 5F9 was GFDILTA (non-overlapping region of Pt9a and Pt9b, rP2-I2, 137–143 aa). Our results confirmed that the epitope recognized by 5F9 resides in Pt9a: GFDILTASSDSAINYT (rP2-I2, 137–152 aa). mAb 2B8 reacted with Pt17, and the recognized epitope was IDFVRTGARFDVTPKSGVYGNYSY (rP2-I2, 257–280 aa). mAbs 1E6, 2C11, and 6E8 reacted with Pt19 and Pt19a, and the epitope recognized was the overlapping region YKLHKQVVTFVE (rP2-I2, 301–312 aa).

The GS-mAbs of genotype I 1A10 reacted with Pt5, but not with Pt5-I3 and Pt5-II, which are peptides in the same region (65–88 aa) of the rP2-I3 and rP2-II proteins, respectively. However, the GS-mAbs of genotype I 2F1 reacted with Pt5-II, but not with Pt5-I3 and Pt5. We then synthesized the truncated peptides, Pt5a and Pt5b, based on the mutation sites in Pt5, and found that neither 1A10 nor 2F1 reacted with these peptides. These results confirmed that the epitope recognized by 1A10 resides in Pt5: YETRLGRNSKNDAGWGDVTTDEAY, while the epitope recognized by 2F1 resides in Pt5-II: YETRLGSGSKNAAKWGDVTTDEAY.

In addition, The GS-mAbs of genotype I 2C5 reacted with Pt11 and Pt11a but not with the truncated peptides Pt11b and Pt11c; therefore, the epitope recognized was the overlapping region of Pt11 and Pt11a, NYNVANERDNKGEVKVDSTKS (rP2-I2, 164–184 aa). Then, Pt11a-II was synthesized (the homologous region of the rP2-II protein). We found that the GS-mAbs of genotype II 5F1 reacted with this peptide, indicating that the epitope recognized by 5F1 resided in the Pt11-II, NYNVANEREKADVKVDSIKS (rP2-II, 180–199 aa).

### 3.4 Multiple alignment of epitope sequence

**Figure 4:**
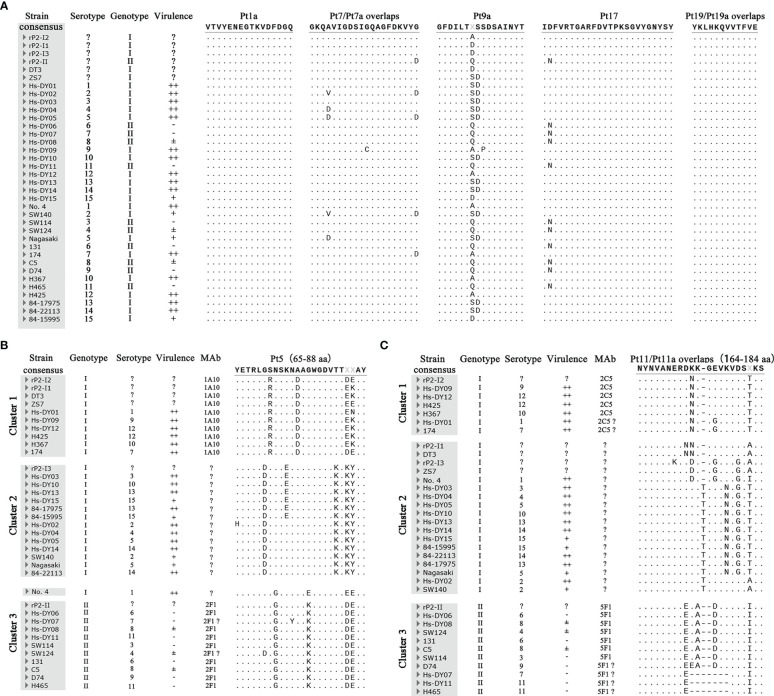
Multiple alignment results of epitope sequence. **(A)** The comparison results of genotype common (GC) epitopes. **(B)** The comparison results of Pt5. **(C)** The comparison results of Pt11/Pt11a. Hs-DY01–Hs-DY15 strains in the picture is the representative strain of the 15 serovars of (*H*) *parasuis* from China. Strains No. 4 84–15995 are the representative strains of the 15 international serovars of (*H*) *parasuis*. In the virulence column, “++” means strong toxicity, which can cause obvious lesions and experimental animal death; “+” means medium toxicity, which can cause obvious lesions but does not cause death; “±” means weak toxicity, causing mild symptoms and transient infection; “-” means no clinical symptoms. “?” means indeterminate.

We compared the sequences of the 36 OmpP2 proteins and showed that the consistency of all sequences was 85.62%, and there was significant heterogeneity between the two genotypes. The epitope sequence alignment results were analyzed in combination with the ELISA results (**Figure 4**).

In terms of the GC-mAbs, the epitopes VTVYENEGTKVDFDGQ (rP2-I2, 1–16 aa) and YKLHKQVVTFVE (rP2-I2, 301–312 aa), recognized by mAbs 1D2/2E2 and 2C11/1E6/6E8, were completely conserved in all OmpP2 sequences. Thus, these two epitopes were GC epitopes. Epitope GFDILTASSDSAINYT (rP2-I2, 137–152 aa), recognized by mAb 5F9, had several amino acid mutations, but these mutations were present within the antigens that reacted with mAb 5F9, including rP2-I1, rP2-I3, rP2-II, and OmpP2 of the DT3 and ZS7 strains. These results showed that the mutations were not at the pivotal amino acids, that all mutation sequences were linear epitopes recognized by mAb 5F9, and that they were also GC epitopes. Similarly, the epitope IDFVRTGARFDVTPKSGVYGNYSY (rP2-I2, 257–280 aa) recognized by mAb 2B8, which had a mutation at N-258 within genotype II proteins, which did not affect its reactivity with mAb 2B8. Thus, these epitopes were GC epitopes. The sequence polymorphism of the epitope GKQAVIGDSIGQAGFDKVYG (rP2-I2, 101–120 aa) recognized by mAb 3A3 was more complicated. Although mutation at D-120 did not affect binding of this peptide to mAbs, we failed to rule out the effect of mutations at aa 104 and 111 on epitope integrity. Multiple alignment revealed that 83.33% (30/36) OmpP2 proteins contained epitopes in this region that could react with mAb 3A3, including genotype I and II proteins; therefore, this epitope was likely to be a GC epitope.

In contrast, the sequences of Pt5 (65–88 aa) and Pt11/Pt11a (164–184 aa), which reacted with GS-mAb, could be divided into three clusters. Except for the Pt5 region of strain no. 4, the two regions of all OmpP2 proteins had obvious genotype specificity; clusters 1 and 2 were genotype I, and cluster 3 was genotype II. mAb 1A10 reacted specifically with the epitopes of rP2-I1, rP2-I2, DT3, and ZS7 in cluster 1, indicating that aa 85 and 86, which had high-frequency mutations, were not pivotal amino acids, and that mutations at these residues did not change the integrity of the epitopes. Therefore, mAb 1A10 interacted specifically with and identifies all OmpP2 proteins in cluster 1. In addition, mAb 1A10 could not recognize rP2-I3 and rP2-II, indicating that mutations at aa 70, 71, 72, 74, 76, 78, and 83 changed the integrity of the epitope, and that these may be pivotal amino acids in the epitope. Therefore, mAb 1A10 could not recognize the sequences of cluster 2 and cluster 3. The epitope YETRLGRNSKNDAGWGDVTT**XX**AY was the GS epitope of cluster 1 of genotype I. Similarly, mAb 2F1 only recognized the proteins of genotype II in cluster 3, but not those in clusters 1 and 2; thus, epitope YETRLGSGSKNAAKWGDVTT**XX**AY was the GS epitope of genotype II. Multiple alignments showed that the epitope Pt11/Pt11a NYNVANERDNKGEVKVDSTKS, recognized by mAb 2C5, was not unique to rP2-I2, and four OmpP2 proteins from representative strains, such as Hs-DY09, also had the same epitope. This epitope was thus a GS epitope belonging to cluster 1 and was significantly different from those in clusters 2 and 3. In contrast, the epitope Pt11a-II NYNVANEREKADVKVDSIKS, recognized by mAb 5F1, was a GS epitope that belonged only to cluster 3 and genotype II. These results illustrated that the pivotal amino acids of the Pt11/Pt11a epitope region contain abundant polymorphisms.

### 3.5 Peptide-ELISA with animal serum

In the peptide-ELISA analysis of mouse serum (**Table 2**), Pt1/Pt1a and Pt9/Pt9a epitopes reacted with mouse serum from both the rP2-I2 and rP2-II immune groups, whereas other epitopes were only exempted in rP2-I2 or rP2-II. The antibody titers of different epitopes showed visible differences in mouse serum; the highest antibody titer of 51,200 of anti-Pt1/P1a was observed in the rP2-II immunization group. This was higher than that of the injected antigen rP2-II (25,600), and 50 in the rP2-I2 group. The antibody titer of anti-Pt5 was 25,600 (rP2-I2 immunization group), whereas the antibody titers of the remaining epitopes ranged between 50 and 3,200. ELISA results showed that anti-Pt14 (THDDYKSGSVNKKDKDGVYFGLKY, rP2-I2, 209–232 aa) antibody was present in the serum of rP2-I2 immunized mice, with a titer of 800, while anti-Pt22 (TYKVDESITVNNTQGTFKYSAPQE, rP2-II, 129–152 aa) antibodies were present in the serum of rP2-II immunized mice, and with a titer of 3,200, indicating that Pt14 and Pt22 may also be epitopes.

**Table 2:** Serum reactivity against epitopes in ELISA.

Peptide/Protein	Antibody titers in rat sera		Reactivity of peptides to porcine sera					
	rP2-I2 injection	rP2-II injection	(a) True positive	(b) False positive	(c) False negative	(d) True negative	Positive rate(%)	Agreement (%)
Group A								
Pt1/Pt1a	50	51200	41	36	27	83	41.18	66.31
Pt5	25600	< 50	3	0	65	119	1.6	65.24
Pt5-I3	< 50	< 50	17	4	51	115	11.23	70.59
Pt5-II	< 50	3200	20	7	48	112	14.44	70.59
Pt7/Pt7a	50	< 50	38	26	30	93	34.22	70.05
Pt9/Pt9a	400	200	12	8	56	111	10.7	65.78
Pt11/Pt11a	800	< 50	12	4	56	115	8.56	67.91
Pt11a-II	< 50	400	46	40	22	79	45.99	66.84
Pt17	50	< 50	49	34	19	85	44.39	71.66
Pt19/Pt19a	3200	< 50	49	32	19	87	43.32	72.73
Group B								
Pt14	800	< 50	31	12	37	107	22.99	73.80
Pt22	< 50	3200	8	3	60	116	5.88	66.31
**Total (group A and B)**			68	58	0	61	67.38	67.91
Group C								
rP2-I2	51200	12800	51	37	17	82	47.06	71.12
rP2-II	6400	25600	62	65	6	54	67.91	62.03
**Total (group C)**			63	66	5	53	68.98	62.03
Group D								
Pt4	< 50	< 50	12	–	8	–	60	
Pt15	< 50	< 50	16	–	4	–	80	
Pt21	< 50	< 50	16	–	4	–	80	

Reactivity of epitopes in swine and mouse sera was tested. A total of 187 swine serum samples were tested by peptide enzyme-linked immunosorbent assays (ELISA) using epitope peptides, and the results were compared with those of a commercial *H. parasuis* antibody ELISA kit. Positive rate = (a + b)/(a + b + c + d); agreement = (a + d)/(a + b + c + d).

In the peptide-ELISA analysis of sera from naturally infected pigs (**Table 2**), positive swine sera could be screened for all epitopes, but the seroprevalence differed markedly among the epitopes. The highest positivity rates were 41.18%, 34.22%, 45.99%, 44.39%, and 43.42% for Pt1/Pt1a, Pt7a, Pt11a-II, Pt17, and Pt19/Pt19a, respectively. The lowest positivity rate was 1.6% for Pt5, and that for the other epitopes was between 5.88% and 22.99%. The overall positivity rate of peptide ELISA for all epitopes and swine serum was 67.38%, similar to the positivity rate (68.98%) for the two recombinant proteins (rP2-I2, rP2-II). Comparative analysis with commercial kits showed that the agreement of each epitope exceeded 65%, with highest being 73.8% for Pt14. The overall agreement between all the epitopes was 67.91%, which was slightly higher than that between the two recombinant proteins (rP2-I2 and rP2-II).

In addition, ELISA for the 20 double-positive serum samples and the remaining peptides showed that the positivity rate of Pt4, Pt15, and Pt21 reached 60%–80%, indicating that these were highly likely to be epitopes. However, the titers of the three peptides were below 50 in mouse serum.

### 3.6 Expression level of proinflammatory cytokines after peptides of OmpP2 protein stimulate PAMs

**Figure 5:**
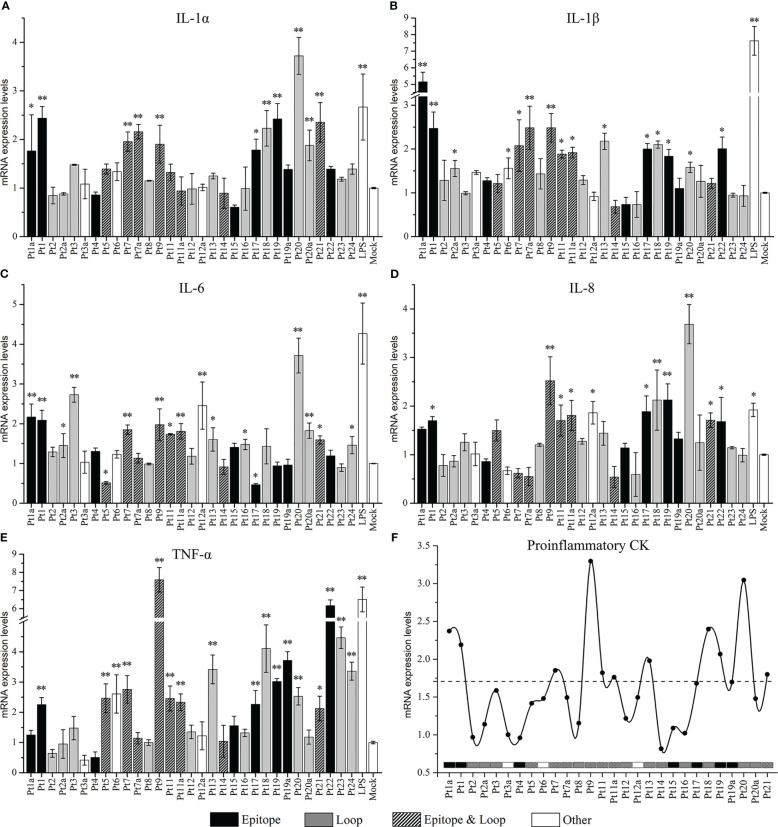
The mRNA expression levels of proinflammatory cytokines induced in pulmonary alveolar macrophages (PAMs) by stimulation with overlapping peptides of OmpP2 protein. PAMs were stimulated with 130 nmol/mL peptides for 12 h. The mRNA expression levels of *IL-1α* **(A)**, *IL-1β* **(B)**, *IL-6* **(C)**, *IL-8* **(D)**, and *TNF-α* **(E)** were detected by real-time polymerase chain reaction. The untreated cells (cultured in serum-free Dulbecco’s modified Eagle’s medium) were used as a mock control. Values are presented as the mean ± SD of three independent experiments. Data were analyzed using one-way analysis of variance, * means *p* < 0.05, ** means *p* < 0.001 compared with the mock control. **(F)** Line graph based on the mean of the mRNA expression levels of *IL-1α, IL-1β, IL-6, IL-8*, and *TNF-α*. The dotted line is the threshold based on the mean of the minimum upregulation level of each cytokine. “Epitope” is the peptide containing only epitopes, “Loop” is the peptide containing only loop, “Epitope & Loop” is the peptide with overlapping epitope and loop, and “Other” is the peptide other than the above three cases.

We compared the mRNA expression levels of *IL-1α, IL-1β, IL-6, IL-8*, and *TNF-α* between peptide-stimulated cells (experimental group) and unstimulated cells (mock group). The results are shown in **Figure 5**. The mRNA expression levels of most cytokines were upregulated (*p* < 0.05) after peptide stimulation, except for that of *IL-6*, which was downregulated by Pt5 and Pt17 stimulation (0.51- and 0.47-fold, respectively, compared to the mock control). The extent of significantly upregulated ranges (compared with mock control, *p* < 0.05) in mRNA expression levels of proinflammatory cytokines were 1.76- to 3.42-fold for *IL-1α*, 1.56- to 5.15-fold for *IL-1β*, 1.45- to 3.71-fold for *IL-6*, 1.68- to 3.69-fold for *IL-8*, and 2.13- to 7.59-fold for *TNF-α*. The highest values were observed upon stimulation with Pt20 (*IL-1α*), Pt1a (*IL-1β*), Pt20 (*IL-6*), Pt20 (*IL-8*), and Pt9 (*TNF-α*) stimulation. Among the peptides producing significant differences in proinflammatory cytokines, 72.73% (8/11, *IL-1α*), 66.67% (10/15, *IL-1β*), 52.94% (8/17, *IL-6*), 72.73% (8/11, *IL-8*), and 64.71% (11/17, *TNF-α*) were epitopes; 63.64% (7/11, *IL-1α*); 53.33% (8/15, *IL-1β*), 70.59% (12/17, *IL-6*), 54.55% (6/11, *IL-8*), 58.82% (10/17, *TNF-α*) were loops; and 36.36% (4//11, *IL-1α*), 33.33% (5/15, *IL-1β*), 35.29% (6/17, *IL-6*), 36.36% (4/11, *IL-8*), 35.29% (6/17, *TNF-α*) were peptides with overlapping epitopes and loops (epitope & loop). Combined analysis of the five proinflammatory cytokines showed that 64.79% (46/71) of the peptides with significant differences were epitopes, 60.56% (43/71) were loops, and 35.21% (25/71) were both epitopes and loops. Although most of the peptides with significant differences in proinflammatory cytokines were epitopes and/or loops, there was no significant difference in the average mRNA expression levels of various cytokines between the epitope and non-epitope groups, or the loop and non-loop groups (**Figure S1**).

The average mRNA expression levels were calculated from five proinflammatory cytokines in each peptide, and a line diagram was drawn with the mean of the minimum upregulation level (significant upregulation compared to the mock control, *p* < 0.05) of each cytokine as the threshold (**Figure 5**). The results showed that the mean values of Pt1, Pt1a, Pt7, Pt9, Pt11, Pt11a, Pt13, Pt18, Pt19, Pt20, and Pt21 exceeded the threshold, and the corresponding amino acid sites were 1–24 aa, 97–120 aa, 129–152 aa, 161–190 aa, 193–216 aa, and 273–341 aa in rP2-I2, which were localized within linear B cell epitopes and loops. These peptides could induce mRNA expression of most inflammatory cytokines. Among them, Pt1, Pt9, and Pt20 significantly induced the mRNA expression of all five inflammatory cytokines (*p* < 0.05) most pronouncedly. In addition, although Pt17 did not exceed the threshold, it affected the expression of five inflammatory cytokines: while it stimulated PAMs to upregulate the expression of *IL-1α*, *IL-1β*, *IL-8* and *TNF-α*, it downregulated the expression of *IL-6* (*p* < 0.05).

**Figure 6:**
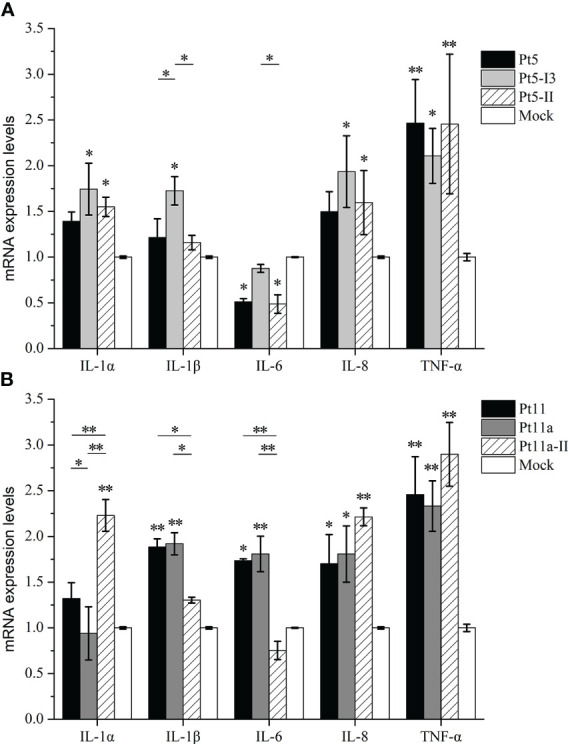
Effect of genotype-specific (GS) epitope peptides on the mRNA expression levels of proinflammatory cytokines. We analyzed the effects of different homologous GS epitopes between two genotypes on proinflammatory cytokine mRNA levels. **(A)** The mRNA expression levels of proinflammatory cytokines in PAMs stimulated with Pt5, Pt5-I3, or Pt5-II. **(B)** The mRNA expression levels of proinflammatory cytokines in PAMs stimulated with Pt11, Pt11a, and Pt11a-II. Untreated cells (cultured in serum-free DMEM) were used as a mock control. Values are presented as the mean ± SD of three independent experiments. Data were analyzed using one-way analysis of variance, * means *p* < 0.05, ** means *p* < 0.001 compared with the mock control.

Finally, we analyzed the effects of different homologous GS epitopes between the two genotypes on proinflammatory cytokines (**Figure 6**). After Pt5-I3 stimulation, the mRNA expression levels of *IL-1α*, *IL-1β*, *IL-8*, and *TNF-α* were significantly upregulated (*p* < 0.05), while *IL-6* levels remained unchanged (*p* > 0.05). Pt5-II could significantly upregulate the levels of *IL-1α*, *IL-8*, *TNF-α*, and significantly downregulate *IL-6* levels (*p* < 0.05). Pt5-I2 could significantly upregulate *TNF-α* and significantly downregulate *IL-6* levels (*p* < 0.05). The comparison among homologous GS epitopes showed that there was no significant difference between Pt5 and Pt5-II (*p* > 0.05), but the induced expression level of *IL-1β* was significantly lower than that of Pt5-I3 (*p* < 0.05). Furthermore, Pt5-II can significantly inhibit the expression of *IL-6* compared with Pt5-I3 (*p* < 0.05). Taken together, these results illustrate that the proinflammatory activities of Pt5-I3 were stronger than those of Pt5 and Pt5-II. Similarly, the expression levels of *IL-1β*, *IL-6*, *IL-8*, and *TNF-α* were significantly upregulated after Pt11 and Pt11a stimulation (*p* < 0.05), while only those of *IL-1α*, *IL-8*, and *TNF-α* were significantly upregulated after Pt11a-II stimulation (*p* < 0.05). Comparison among the three homologous GS epitopes showed that the mRNA expression levels of *IL-1β* and *IL-6* after Pt11 and Pt11a stimulation were significantly higher than those after Pt11a-II stimulation, but the level of *IL-1α* was lower than that after Pt11a-II stimulation (*p* < 0.05).

## 4 Discussion

As a commensal organism and pathogen, different *H. parasuis* strains show varying degrees of virulence and heterogeneity; therefore, distinguishing between non-virulent and virulent strains is very important for the prevention, control, and diagnosis of the disease. The identification of virulence factors is the most direct and effective way to distinguish bacterial virulence. OmpP2 is one of the main virulence factors of *H. parasuis*, associated with the growth, colonization, adhesion, invasion, serum resistance, phagocytosis resistance, and proinflammatory activity of the organism (Zhang et al., 2012a; Zhang et al., 2012b; Zhou et al., 2014; Shen et al., 2019). Mullins et al. first found that *ompP2* showed high heterogeneity among different strains, based on sequencing (Mullins et al., 2009). Moreover, Zhao and Li obtained the same results by analyzing clinically isolated strains from China and divided *ompP2* into genotypes I and II according to heterogeneity (Zhao et al., 2010; Li et al., 2012). OmpP2 of genotype I displayed more cytotoxicity and serum resistance, indicating a relationship between the genotype and the virulence of the strain (Cerda-Cuellar and Aragon, 2008; Zhao et al., 2010). We analyzed the OmpP2 sequences of representative strains of different serotypes and found that apart from the two known gene insertions, genotype-specific mutations were found at multiple sites between genotypes I and II, mainly distributed in the loop region, such as Pt5, Pt9, Pt11/Pt11a, and Pt17 (**Figure 4**). We then traced the virulence of each representative strain and found that the OmpP2 sequences of all strains with strong and medium toxicity belonged to genotype I, whereas the strains with weak toxicity and non-virulence belonged to genotype II. Therefore, it is possible to distinguish the virulence of the strains according to the OmpP2 genotype. However, as a caveat, it should be noted that the initial study showed that strain 174 infected SPF pigs through intraperitoneal injection without obvious symptoms; therefore, it was considered a nonvirulent strain (Kielstein and Rapp-Gabrielson, 1992). On the other hand, recent studies showed that strain 174 could cause obvious symptoms and even death after infecting conventional and colostrum-deprived pigs through intratracheal injection, indicating that it was a virulent strain (Guizzo et al., 2018). This result was confirmed by pathotyping-PCR (Schuwerk et al., 2020).

Insertion of the OmpP2 sequence changes the antigen-determinant cluster exposed to the membrane (Mullins et al., 2009; Zhao et al., 2010). This phenomenon has also been observed in the porins of most Gram-negative bacteria (Brunham et al., 1993). In the present study, GS-mAbs against different genotype proteins appeared after immunization with rOmpP2 of different genotypes (rP2-I2 and rP2-II), which was in agreement with previous findings. Through screening of the 32 mAbs obtained in this study, we identified nine epitopes, including GC epitopes Pt7/Pt7a, Pt9a, Pt17, and Pt19; GS epitopes of genotype I: Pt5 and Pt11/Pt11a; and GS epitopes of genotype II: Pt5-II and Pt11a-II. As the next step, we will establish an indirect ELISA method based on using GS epitopes as coated antigens to detect antibodies against proteins of different genotypes in serum. However, GS-mAbs can be used as coated antibodies and GS-mAbs as test antibodies to establish a double-antibody sandwich ELISA to detect bacterial antigens of different genotypes. Our results showed that it was possible to differentiate the OmpP2 protein genotype, and the virulence of the strains was ascertained. According to the ELISA results of clinical swine serum used in this study, the agreement between the epitope peptide-ELISA and the commercially available kit was only 65.24%–73.8%. The commercial kit involves coating with outer membrane proteins from a virulent strain, and the direction of optimization is to detect specific antibodies to virulent strains; therefore, the agreement with peptide-ELISA was unsatisfactory. The most suitable antigen coating process and judgment standard for the established peptide-ELISA method need to be optimized in future.

The prevention of *H. parasuis* mainly relies on vaccines. Currently, most commercial *H. parasuis* vaccines use inactivated organisms and are serovar-specific, with limited cross-protection against different virulent serovars (Liu et al., 2016). Studies have shown that the antigenicity of different serotypes, or strains of the same serotype, may vary (Liu et al., 2016; Costa-Hurtado et al., 2020). Therefore, few managers have taken the initiative to inject inactivated vaccines for *H. parasuis* prevention because of the poor efficacy of single-serotype inactivated vaccines. To address this issue, scientists have focused on the development of subunit vaccines, which are mainly composed of specific dominant antigens or multiple epitopes of different serotypes, which can lead to an immune response towards common epitopes present in virulent strains, to provide cross-protective effects, while avoiding affecting non-virulent *H. parasuis* in nasal microecology (Costa-Hurtado et al., 2020). OmpP2 is well-suited for producing subunit vaccines. First, mouse experiments showed that virulent *H. parasuis* infection can quickly induce the production of high-efficiency anti-OmpP2 antibodies, effectively kill bacteria in the presence of complement, inhibit bacterial growth, and improve the survival rate of mice (Zhou et al., 2009a). In the present study, the titers of immunized mice injected with rP2-I2 or rP2-II reached 51,200 and 25,600, respectively, indicating that OmpP2 proteins of different genotypes could induce high titers of specific antibodies. Therefore, OmpP2 is a dominant antigen with strong immunogenicity that is related to protective immunity against *H. parasuis* infection. Second, our study showed that 84.38% of the mAbs recognized the linear B-cell epitopes of OmpP2, indicating that OmpP2 contains major linear epitopes, and that most epitopes, such as Pt1/Pt1a, Pt7/P t7a, Pt17, and Pt19/Pt19, had a high positive rate in clinical samples. These epitopes are suitable for assembling multi-epitope vaccines. Finally, the key to subunit vaccines is the screening and identification of specific epitopes of virulent strains, such as F6 fragments exposed on the surface of the VtaA protein (Correa-Fiz et al., 2017), loop fragments of neuraminidase (Bregon-Villahoz et al., 2017), and specific epitope fragments of TbpA and TbpB (Frandoloso et al., 2011), which are considered ideal candidates for subunit vaccines. Our results also identified specific epitopes of OmpP2 of virulent *H. parasuis*, including Pt5 and Pt11/Pt11a. Although the amino acid sequences of these epitopes are not conserved in genotype I, a multi-epitope vaccine can be produced by combining the differential epitopes of genotype I, which is an advantage of subunit vaccines.

In addition to antigen factors, attention should be paid to the species of experimental animals used in the development of subunit vaccines. At present, most of the above candidate molecules have been verified in mice, but not in the natural host of *H. parasuis*. However, the immunological effects of the same immunogen may vary among species. For example, pigs immunized with VtaA did not produce antibodies against the F6 fragments (Correa-Fiz et al., 2017). In addition, some studies used rOmpP2 to immunize colostrum-deprived pigs, showing that it could only delay death in experimental pigs after a challenge experiment with lethal doses of strains, and not allow the hosts to survive as observed in the corresponding mouse experiment (Alvarez-Estrada et al., 2018). Although sera positive for all epitopes screened by mouse-derived mAbs were present in swine herds, there were some differences between the two species. For example, the GC epitope Pt9/Pt9a showed a moderate antibody titer in both mouse immunization groups, but the positive rate in swine serum was significantly lower than that of other GC epitopes. In contrast, the antibody titer in mouse serum was very low after immunization with the GC epitope Pt17, whereas the positivity rate in swine serum was higher than that of other GC epitopes. The largest difference was that epitopes Pt4, Pt15, and Pt21, which could be recognized by *H. parasuis* seropositive samples, did not induce antibodies in mice. Therefore, the selection of new subunit vaccine candidates should fully consider the responses of natural hosts and avoid omissions and misselections.

PAMs are the primary line of defense against *H. parasuis* (Olvera et al., 2009; Macedo et al., 2015). *In vitro* experiments showed that nonvirulent strains were efficiently phagocytosed by PAMs, whereas virulent strains were resistant to phagocytosis (Olvera et al., 2009). Therefore, for virulent *H. parasuis*, escape is the first step in inducing Glässer disease. Previous studies have shown that antibody-mediated opsonization can facilitate the phagocytosis of virulent *H. parasuis* by PAMs, which prevents the development of the disease in the early stages of infection (Matiaskova et al., 2021). In the present study, we found that there were six linear B-cell epitopes in the loop region of OmpP2 that were exposed to the surface of bacteria, distributed in loop2 (Pt5, Pt5-II), loop3 (Pt7/Pt7a, Pt9), loop4 (Pt11/Pt11a), loop5 (Pt14), and loop8 (Pt21), suggesting that these epitopes and corresponding antibodies may participate in the opsonization and phagocytosis resistant of *H. parasuis*. Serum resistance is another major mechanism by which *H. parasuis* escapes. OmpP2 of both genotypes I and II participated in the serum resistance of *H. parasuis*, and genotype I OmpP2 exhibited significantly increased resistance to complement killing. However, the specific mechanism remains unclear (Cerda-Cuellar and Aragon, 2008). Recent studies have confirmed the existence of a binding site (K-278, K-280, D-281) of complement C1q on loop7 of porin OMPK36 of *Klebsiella pneumoniae*, which determines its serum sensitivity (Alberti et al., 1995). The conserved motif of the C1q binding site is a string-like arrangement of one negative and two positive side chains, which are generally distributed at the connection between the loop and the β-strands or in the loop region (Alberti et al., 1995; Alberti et al., 1996). We found that OmpP2 may have multiple suspected binding sites for C1q and is located close to and even overlapping with B cell epitopes. For example, 81-DVKTK-85 in GS epitope Pt5-I3, which was unique to cluster 2 of genotype I, 135-KKGFD-139 in Pt9, which was unique to genotype I, 317-KNKD-320 close to Pt21, etc. (aa numbers refer to rP2-I2). Next, we verified whether these epitopes are binding sites of C1q and explored the effects of these epitopes as well as the serum resistance of their antibodies.

The escaped *H. parasuis* can invade the endothelial cells of the tissue, stimulate cells to secrete proinflammatory cytokines, and induce macrophage and granulocyte recruitment, causing Glässer disease, which is characterized by severe systemic inflammation (Costa-Hurtado and Aragon, 2013). As the main virulence factor, OmpP2 participates in all of the processes mentioned above, particularly in the induction of an inflammatory response (Zhao et al., 2010; Zhang et al., 2012a; Zhang et al., 2012b; Costa-Hurtado and Aragon, 2013; Zhang et al., 2014; Zhou et al., 2014). Previous studies showed that the OmpP2 of the virulent strains could activate NF-κB and MAPK signaling pathways to induce the expression of inflammatory cytokines, such as IL-1α, IL-1β, IL-6, IL-8, IL-17, IL-23, CCL-4, and CCL-5. Further research revealed that the eight loops of OmpP2 upregulate the expression of proinflammatory cytokines. Moreover, the upregulated effect of loop7 and loop8 of OmpP2 was the most obvious, and was almost equivalent to that of OmpP2 (Zhou et al., 2019). We also found that most peptides overlapping with loop3, loop4, loop5, loop7, and loop8 could also upregulate the mRNA expression levels of proinflammatory cytokines (*IL-1α, IL-1β, IL-6, IL-8*, and *TNF-α*). In particular, Pt18, overlapping with loop7, and Pt20, overlapping with loop8, showed a higher degree of upregulation than most other peptides, matching the results of previous studies. However, the upregulatory effect of Pt20a, which includes a complete loop8, was not consistent with previous research on this loop. We speculate that this may be due to the abundance of hydrophobic amino acids at both ends of Pt20a. In addition to these loops, our study found that linear B-cell epitopes could stimulate PAMs to upregulate the mRNA expression levels of proinflammatory cytokines, including Pt7/Pt7a, Pt9, Pt11/Pt11a, and Pt21, which overlapped with loops, and epitopes Pt1/Pt1a, Pt17, and Pt19/Pt19a, which did not. Taken together, Pt1, Pt1a, Pt7, Pt9, Pt11, Pt11a, Pt13, Pt18, Pt19, Pt20, and Pt21 (rP2-I2: 1–24 aa, 97–120 aa, 129–152 aa, 161–190 aa, 193–216 aa, 273–341 aa) significantly stimulated PAMs to upregulate the mRNA expression levels of most proinflammatory cytokines and all overlapped with B-cell epitopes or loops. This suggests that these epitopes and loop peptides may be the virulence-related sites of OmpP2. Among them, Pt1, Pt9, and Pt20 upregulated the mRNA expression levels of five proinflammatory cytokines (*IL-1α, IL-1β, IL-6, IL-8*, and *TNF-α*) in PAMs (*p* < 0.05), indicating that they are the primary virulence-related sites of OmpP2. However, it should be noted that our subgroup analysis found that there was no difference in the mRNA expression levels of *IL-1α, IL-1β, IL-6, IL-8*, and *TNF-α* between epitope and non-epitope peptides (*p* > 0.05), or loop and non-loop peptides (*p* > 0.05). Moreover, epitopes not only upregulated the levels of the mRNA expression levels of proinflammatory cytokines: for example, epitope Pt5, Pt5-II, and Pt17 could significantly downregulate the level of *IL-6* mRNA (*p* < 0.05).

Mutations in multiple amino acids in *H. influenzae* loop7 of OmpP2 can lead to a reduction in the expression of cytokines, followed by a decrease in proinflammatory activity (Galdiero et al., 2006). Our study found that there were multiple amino acid mutations in the GS epitopes of OmpP2, leading to differences in the mRNA expression levels of proinflammatory cytokines when these peptides were used to stimulate PAMs. For instance, significantly differences in the mRNA levels of *IL-1β* and *IL-6* could be observed between stimulation with Pt5-I3 and Pt5-II (*p* < 0.05), and in the levels of *IL-1α*, *IL-1β*, and *IL-6* between stimulation with Pt-11/Pt-11a and Pt11a-II (*p* < 0.05). The proinflammatory cytokines that were differentially expressed in both homologous GS epitopes groups were *IL-1β* and *IL-6*, and both were expressed at lower levels in response to genotype II epitope than genotype I epitope stimulation (*p* < 0.05). These results indicated that the GS epitopes of OmpP2 may affect the virulence of *H. parasuis* by affecting the expression of proinflammatory cytokines. It was also necessary to compare all the different GS epitopes (mutant GS epitopes) and loops of genotypes I and II.

In conclusion, this study preliminarily determined the distribution of linear B-cells epitopes of OmpP2 and explored their role in *H. parasuis*-induced inflammation. This provides a strong basis for exploring the host’s anti-infection immune mechanism, establishing a method to distinguish the pathogenicity of strains, and screening candidate peptides for subunit vaccines that can promote the prevention and treatment of Glässer disease.

## Data availability statement

The datasets presented in this study can be found in online repositories. The names of the repository/repositories and accession number(s) can be found in the article/**Supplementary Material**.

## Ethics statement

The animal study was reviewed and approved by Institutional Animal Care and Use Committee (IACUC) of Shaoguan University.

## Author contributions

JW, WN, and GP designed and planed the study. JW, WN, HH, JH implement experiment. JW, HH and JH contributed to data collation and analysis. CX, ZX contributed to interpretation. JW completed the drafting of the manuscript. WN, GP, ZX revised the manuscript. WN, CX supervised the research. All authors contributed to the article and approved the submitted version.
