# Supplementary material for: Screening of linear B-cell epitopes and its proinflammatory activities of Haemophilus parasuis outer membrane protein P2
Source: Front Cell Infect Microbiol. 2023 May 3;13:1192651. doi: 10.3389/fcimb.2023.1192651 (PMC10189045; doi:10.3389/fcimb.2023.1192651)
Supplement: Supplementary file 1 [file DataSheet_1.docx]

Supplementary Material

**Screening of linear B-cell epitopes and its** **Proinflammatory Activities** **of *Haemophilus parasuis* outer membrane protein P2**

**Jingbo Wu^1^****^,2,3^, Wenjin Nan^1,4*^, Guoliang Peng^1,3^**, **Honghui Hu^1,3^, Chongbo Xu^1,2^, Jianqiang Huang^1,2^, Zhengzhong Xiao^1,2^**

*** Correspondence:** Wenjin Nan: [nanwenjin@sgu.edu.cn](mailto:nanwenjin@sgu.edu.cn)

**Supplementary Table 1**  Sequences of the PCR primers of proinflammatory cytokines

| Primers | Sequence | Product length (bp) | Annealing temperature | Reference |
| --- | --- | --- | --- | --- |
| IL-1α/F | GAAGAAGAGACGGTTGAG | 109 | 58℃ | Zhou et al, 2019 |
| IL-1α/R | GCTGTATGTTGCTGATCT |  |  |  |
| IL-1β/F | ACCTGGACCTTGGTTCTCTG | 83 | 58℃ |  |
| IL-1β/R | CATCTGCCTGATGCTCTTGT |  |  |  |
| IL-6/F | AATCCAGACAAAGCCACCAC | 79 | 60℃ |  |
| IL-6/R | TCCACTCGTTCTGTGACTGC |  |  |  |
| IL-8/F | TAGGACCAGAGCCAGGAAGA | 92 | 58℃ |  |
| IL-8/R | AGCAGGAAAACTGCCAAGAA |  |  |  |
| TNF-α/F | CCACCAACGTTTTCCTCACT | 82 | 58℃ |  |
| TNF-α/R | TTGATGGCAGAGAGGAGGTT |  |  |  |
| GAPDH/F | CCCCAACGTGTCGGTTGT | 80 | 58℃ |  |
| GAPDH/R | GCTTCACCACCTTCTTGA |  |  |  |


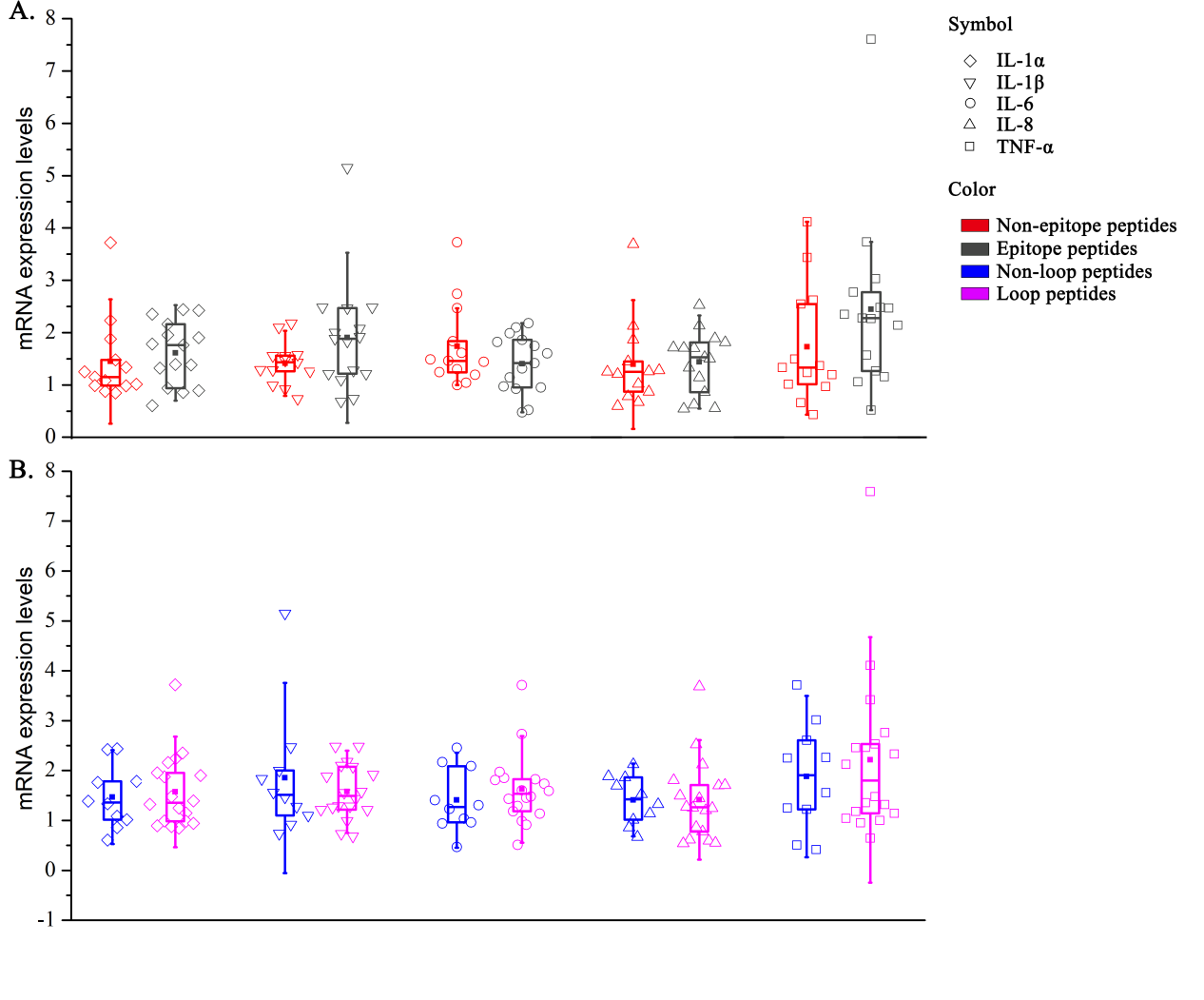


**Supplementary Figure 1.** Effects of epitope peptides and loop peptides on the mRNA expression levels of proinflammatory cytokines. Overlapping peptides were divided into epitope group and non-epitope group, loop group and non-loop group, then the mRNA expression levels of cytokines of each group were compared. Data were analyzed using one-way analysis of variance, showing that there was no significantly difference between the data (*p* > 0.05).
