# Supplementary material for: Screening of linear B-cell epitopes and its proinflammatory activities of Haemophilus parasuis outer membrane protein P2
Source: Front Cell Infect Microbiol. 2023 May 3;13:1192651. doi: 10.3389/fcimb.2023.1192651 (PMC10189045; doi:10.3389/fcimb.2023.1192651)
Supplement: Supplementary file 2 [file DataSheet_2.doc]

Supplementary Material

**Screening of linear B-cell epitopes and its** **Proinflammatory Activities** **of *Haemophilus parasuis* outer membrane protein P2**

**Jingbo Wu^1^****^,2,3^, Wenjin Nan^1,3*^, Guoliang Peng^1,3^**, **Honghui Hu^1,3^, Chongbo Xu^1,2^, Jianqiang Huang^1,2^, Zhengzhong Xiao^1,2^**

*** Correspondence:** Wenjin Nan: [nanwenjin@sgu.edu.cn](mailto:nanwenjin@sgu.edu.cn)

**Supplementary Data: The sequence of OmpP2 of recombinant proteins, clinical isolates and reference strains**

>hps ZS7 GenBank accession numbers OQ693625

ATGGTAACAGTTTATGAAAATGAAGGTACAAAAGTTGATTTTGATGGTCAATTGCGTCTTCTTTTAGAAGAACAAGCCACAAAAGAGAAAGGTCAATCTTCAACAGGTGGTCACACTAACTTAAAGAATAATGGTTCTCGTTTCGGTATTTCTATCAAACATAATATCAATGAGAATCTCTACGGTTTTGGTCGTTATGAGACTCGCCTTGGCCGTAATTCTAAAAATGATGCAGGATGGGGGGATGTTACAACAGAAGAGGCTTACGTCGGTTTAGGCGGCTATGGTCATGAAATTTCTTTTGGTAAACAAGCTGTAATCGGTGATAGCATTGGTCAAGCTGGTTTTGATAAAGTATACGGTGTTGGTAGTACTGGAATTAGAATTAAATATCCAGCAAACTCAGCAGACAAAAAAGGTTTTGATATCCTTACTTCAGATTCTGATTCAGCAATTAACTATACCTATACAGGCATTGAAGGTTTGACGTTAGGTGCTAACTATAATGTTGCAAATGAGCGTGATGATAAGGGAGGCGTAAAAGTAGGTTCTGCTAAATCTGGCTTTGGTTTAGGTGCTAAATACACAGCTAAGATTGCGGAAAGTCAATCTGTAACTGTGGCAGCAGGTTATACTCATGATGACTATAAATCTGGATCTGTTAATAAGAAAGACAAAGATGGTGTATACTTTGGTCTTAAATATGTCAATGCTCCATTTACTGTAGCTGTTGATGGTGGTCATGGTGTTGAAAAAACAGGTAATGTTAAAGAGAAAATTGACTTCGTAAGAACTGGCGCAAGATTTGATGTTACTCCAAAATCTGGCGTGTATGGAAACTACTCTTATGGTACTTACAAAAATAAAGCTTACAAAGCAACAGCTCATCAATTCATGTTAGGTGCAGACTATAAATTACATAAACAAGTTGTTACCTTTGTTGAAGGTCGTTTAATCAAGAACAAAGACAGTAATAACAAAAAAGTTACTGACCAAGCACTTGGTGTTGGTTTACGTGTATTATGG

>hps DT3 GenBank accession numbers OQ693626

ATGGTAACAGTTTATGAAAATGAAGGTACAAAAGTTGATTTTGATGGTCAATTGCGTCTTCTTTTAGAAGAACAAGCCACAAAAGAGAAAGGTCAATCTTCAACACGTGGTCACACTAACTTAAAGAATAATAGTTCTCGTTTCGGTATTTCTATCAAACATAATATCAATGAGAATCTCTACGGTTTTGGTCGTTATGAGACTCGCCTTGGCCGTAATTCTAAAAATGATGCAGGATGGGGGGATGTTACAACAGAAAAGGCTTACGTCGGTTTAGGCGGCTATGGTCATGAAATTTCTTTTGGTAAACAAGCTGTAATCGGTGATAGCATTGGTCAAGCTGGTTTTGATAAAGTATACGGTGTTGGTACTGGTGGAATTAAATATTCAGCAAACAACACAAACAAAAAAGGTTTTGATATCCTTACTGACTCTTCTGATTCAGCAATTAACTATACCTATACAGGCATTGAAGGTTTGACGTTAGGTGCTAACTATAATGTTGCAAATGAGCGTAATAATAAGGGAGAAGTAAAAGTAGATTCTGCTAAATCTGGCTTTGGTTTAGGTGCTGAATACACAGCTAAGATTGCAGAAAGTCAATCTGTAACTGTGGCAGCAGGTTATACTCATGATGACTATAAATCTGGATCTTCTGGATCTGTGTCATTTAATAAGAAAGACAAAGATGGTGTATACTTTGGTCTTAAATATGTCAACGCTCCATTTACTGTAGCTGTTGATGGTGGTCATGGTGTTCTAAAAACAGATAATGTTAAAGAGAAAATTGACTTCGTAAGAACTGGTGCAAGATTTGATGTTACTCCAAAATCTGGCGTGTATGGAAACTACTCTTATGGTACTTACAAAGATAAAGCTTACAAAGCAACAGCTCATCAATTCATGTTAGGTGCAGACTATAAATTACATAAACAAGTTGTTACCTTTGTTGAAGGTCGTTTAATCAAGAACAAAGACAGTAATAACAAAAAAGTTACTGACCAAGCACTTGGTGTTGGTTTACGTGTATTATGG

>hps rP2-I2 GenBank accession numbers OQ693622

ATGGTAACAGTTTATGAAAATGAAGGTACAAAAGTTGATTTTGATGGTCAATTGCGTCTTCTTTTAGAAAAACAAGCCTCAAAAGTGAAAGGTCAATCTTCAACAAGTGGTCACACTGACTTAAAGAATAATGGTTCTCGTTTCGGTATTTCTATCAAACATAATATCAATGAGAATCTCTACGGTTTTGGTCGTTATGAGACTCGCCTTGGCCGTAATTCTAAAAATGATGCAGGATGGGGGGATGTTACAACAGATGAGGCTTACGTCGGTTTAGGCGGCTATGGTCATGAAATTTCTTTTGGTAAACAAGCTGTAATCGGTGATAGCATTGGTCAAGCTGGTTTTGATAAAGTATACGGTGTTGGTACTGGTGGAATTAAATATTCAGCAAACAACACAAACAAAAAAGGTTTTGATATCCTTACTGCATCTTCTGATTCAGCAATTAACTATACCTATACAGGCATTGAAGGTTTGACGTTAGGTGCTAACTATAATGTTGCAAATGAGCGTGATAATAAGGGAGAAGTAAAGGTAGATTCTACTAAATCTGGCTTTGGTTTAGGTGCTAAATACACAGCTAAGATTGCGGAAAGTCAATCTGTAACTGTGGCAGCAGGTTATACTCATGATGACTATAAATCTGGATCTGTTAATAAGAAAGACAAAGATGGTGTATACTTTGGTCTTAAATATGTCAATGCTCCATTTACTGTAGCTGTTGATGGTGGTCATGGTGTTGAAAAAACAGGTAATGTTAAAGAGAAAATTGACTTCGTAAGAACTGGCGCAAGATTTGATGTTACTCCAAAATCTGGCGTGTATGGAAACTACTCTTATGGTACTTACAAAAATAAAGCTTACAAAGCAACAGCTCATCAATTCATGTTAGGTGCAGACTATAAATTACATAAACAAGTTGTTACCTTTGTTGAAGGTCGTTTAATCAAGAACAAAGACAGTAATAACAACAAAGTTACTGACAAAGCACTTGGTGTTGGTTTACGTGTATTATGG

>hps rP2-I1 GenBank accession numbers OQ693621

ATGGTAACAGTTTATGAAAATGAAGGTACAAAAGTTGATTTTGATGGTCAATTGCGTCTTCTTTTAGAAGAACAAGCCACAAAAGAGAAAGGTCAATCTTCAACACGTGGTCACACTAACTTAAAGAATAATAGTTCTCGTTTCGGTATTTCTATCAAACATAATATCAATGAGAATCTCTACGGTTTTGGTCGTTATGAGACTCGCCTTGGCCGTAATTCTAAAAATGATGCAGGATGGGGGGATGTTACAACAGAAAAGGCTTACGTCGGTTTAGGCGGCTATGGTCATGAAATTTCTTTTGGTAAACAAGCTGTAATCGGTGATAGCATTGGTCAAGCTGGTTTTGATAAAGTATACGGTGTTGGTACTGGTGGAATTAAATATTCAGCAAACAACACAAACAAAAAAGGTTTTGATATCCTTACTGACTCTTCTGATTCAGCAATTAACTATACCTATACAGGCATTGAAGGTTTGACGTTAGGTGCTAACTATAATGTTGCAAATGAGCGTAATAATAAGGGAGAAGTAAAAGTAGATTCTGCTAAATCTGGCTTTGGTTTAGGTGCTGAATACACAGCTAAGATTGCAGAAAGTCAATCTGTAACTGTGGCAGCAGGTTATACTCATGATGACTATAAATCTGGATCTTCTGGATCTGTGTCATTTAATAAGAAAGACAAAGATGGTGTATACTTTGGTCTTAAATATGTCAACGCTCCATTTACTGTAGCTGTTGATGGTGGTCATGGTGTTCTAAAAACAGATAATGTTAAAGAGAAAATTGACTTCGTAAGAACTGGTGCAAGATTTGATGTTACTCCAAAATCTGGCGTGTATGGAAACTACTCTTATGGTACTTACAAAGATAAAGCTTACAAAGCAACAGCTCATCAATTCATGTTAGGTGCAGACTATAAATTACATAAACAAGTTGTTACCTTTGTTGAAGGTCGTTTAATCAAGAACAAAGACAGTAATAACAAAAAAGTTACTGACCAAGCACTTGGTGTTGGTTTACGTGTATTATGG

>hps rP2-I3 GenBank accession numbers OQ693623

ATGGTAACAGTTTATGAAAATGAAGGTACAAAAGTTGATTTTGATGGTCAATTGCGTCTTCTTTTAGAAGAACAAGCCACAAAAGAGAAAGGTCAATCTTCAACAGGTGGTCACACTAACTTAAAGAATGATGGTTCTCGTTTCGGTATTTCTATCAAACATAATATCAATGAGAATCTCTACGGTTTTGGTCGTTATGAGACTCGCCTTGACAGTAATTCTGAAAATGCTGCAGGATGGGGCGATGTTAAAACAAAATATGCTTACGTCGGTTTAGGCGGCTATGGTCATGAAATTTCTTTTGGTAAACAAGCTGTAATCGGTGATAGCATTGGTCAAGCTGGTTTTGATAAAGTATACGGTGTTGGTACTGGTGGAATTAAATATTCAGCAAACAACACAAACAAAAAAGGTTTTGATATCCTTACTGACTCTTCTGATTCAGCAATTAACTATACCTATACAGGCATTGAAGGTTTGACGTTAGGTGCTAACTATAATGTTGCAAATAAGCGTGATGATAAGGGAGGCGTAAAAGTAGGTTCTGCTAAATCTGGCTTTGGTTTAGGTGCTGAATGCACAGCTAAGACTGCAGAAAGTCAATCTGTAACTGTGGCAGCAGGTTATACTCATGATGACTATAAATCTGGATCTTCTGGATCTGTGTCATTTAATAAGAAAGACAAAGATGGTGTATACTTTGGTCTTAAATATGTCAACGCTCCATTTACTGTAGCTGTTGATGGTGGTCATGGTGTTCTAAAAACAGATAATGTTAAAGAGAAAATTGACTTCGTAAGAACTGGTGCAAGATTTGATGTTACTCCAAAATCTGGCGTGTATGGAAACTACTCTTATGGTACTTACAAAGATAAAGCTTACAAAGCAACAGCTCATCAATTCATGTTAGGTGCAGACTATAAATTACATAAACAAGTTGTTACCTTTGTTGAAGGTCGTTTAATCAAGAACAAAGACAGTAATAACAAAAAAGTTACTGACCAAGCACTTGGTGTTGGTTTACGTGTATTATGG

>hps rP2-II GenBank accession numbers OQ693624

ATGGTAACAGTTTATGAAAATGAAGGTACAAAAGTTGATTTTGATGGTCAGTTGCGTCTTCTTTTAGAAAAAAAAGCCTCAAAAGATAAAGGTAAATCTTCAACAGATGGTCACACTAACTTAAAGAATAATAGTTCTCGTTTCGGTATTTCTATCAAACATAATATCAATGAGAATCTCTACGGTTTTGGTCGTTATGAGACTCGCCTTGGCAGTGGTTCTAAAAATGCTGCAAAATGGGGTGATGTTACAACAGATGAGGCTTACGTTGGTTTAGGTGGCTATGGTCATGAAATTTCTTTTGGTAAACAAGCTGTAATCGGTGATAGCATTGGTCAAGCTGGTTTTGATAAAGTATACGATGTTGGTACTGGTGGAATTAAATATACATATAAGGTAGATGAGTCTATCACTGTGAACAATACACAGGGTACATTTAAATATTCAGCACCTCAAGAAGGTTTTGATATCCTTACTCAATCTTCTGATTCAGCAATTAACTATACCTATACAGGTATTGAAGGTTTGACGTTAGGTGCTAACTATAATGTTGCAAATGAGCGTGAGAAGGCAGATGTAAAAGTAGATTCTATTAAATCTGGCTTTGGTTTAGGTGCTAAATACACAGCTAAGATTGCAGAAAGTCAATCTGTAACTGTGGCAGCAGGTTATACTCATGATGACTACAAATCTGGATCTGTTCAACTAAAAGGTAAATTTGTTCAAGCAAATGGTACATCTACAGACCATACCTATACAGAGTCATTTAATAAGAAAAACAAAGATGGTGTATACTTTGGTCTTAAATATGTCAACGCTCCATTTACTGTAGCTGTTGATGGTGGTCATGGTGTTGTAAAAACAGATGATGTTAAAGAGAAAATTAACTTCGTAAGAACTGGCGCAAGATTTGATGTTACTCCAAAATCTGGCGTGTATGGAAACTACTCTTATGGTACTTACAAAGTTGAAGATTACAAAGCAACAGCTCATCAATTCATGTTAGGTGCAGACTATAAATTACATAAACAAGTTGTTACCTTTGTTGAAGGTCGTTTAATCAAGAACAAAGACAGTGATAACAACAAAGTTACTGACAAAGCACTTGGTGTTGGTTTACGTGTATTATGG

>gi|284929970|gb|GU323687.1| Haemophilus parasuis strain Hs-DY01 outer membrane protein P2 precursor (ompP2) gene, complete cds

ATGAAAAAAACACTAGTAGCGTTAGCAGTAGCGGCATTTGCAGCATCAGCATCAGCTGTAACAGTTTATGAAAATGAAGGTACAAAAGTTGATTTTGATGGTCAATTGCGTCTTCTTTTAGAAAAACAAGCCTCAAAAGTGAAAGGTCAATCTTCAACAACAGATGGTCACACTAACTTAAAGAATAATAGTTCTCGTTTCGGTATTTCTATCAAACATAATATCAATGAGAATCTCTACGGTTTTGGTCGTTATGAGACTCGCCTTGGCCGTAATTCTAAAAATGATGCAGGATGGGGGGATGTTACAACAGAAAATGCTTACGTCGGTTTAGGCGGCTATGGTCATGAAATTTCTTTTGGTAAACAAGCTGTAATCGGTGATAGCATTGGTCAAGCTGGTTTTGATAAAGTATACGGTGTTGGTACTGGTGGAATTAAATATTCAGCAAACAACACAAACAAAAAAGGTTTTGATATCCTTACTTCAGATTCTGATTCAGCAATTAACTATACCTATACAGGTATTGAAGGTTTGACGTTAGGTGCTAACTATAATGTTGCAAATGAGCGTGATAATAAGGGAGGAGTAAAGGTAGATTCTACTAAATCTGGCTTTGGTTTAGGTGCTAAATACACGGCTAAGATTGCGGAAAGTCAATCTGTAACTGTGGCTGCAGGTTATACTCATGATGACTATAAATCTGGATCTGTTAATAAGAAAGACAAAGATGGTGTATACTTTGGTCTTAAATATGTCAATGCTCCATTTACTGTAGCTGTTGATGGTGGTCATGGTGTTGTAAAAACAGATAATGTTAAAGAGAAAATTGACTTCGTAAGAACTGGCGCAAGATTTGATGTTACTCCAAAATCTGGCGTGTATGGAAACTACTCTTATGGTACTTACAAAGATAAAGCTTACAAAGCAACAGCTCATCAATTCATGTTAGGTGCAGACTATAAATTACATAAACAAGTTGTTACCTTTGTTGAAGGTCGTTTAATCAAGAACAAAGACAGTAATAACAAAAAAGTTACTGACCAAGCACTTGGTGTTGGTTTACGTGTATTATGGTAA

>gi|284929972|gb|GU323688.1| Haemophilus parasuis strain Hs-DY02 outer membrane protein P2 precursor (ompP2) gene, complete cds

ATGAAAAAAACACTAGTAGCGTTAGCAGTAGCGGCATTTGCAGCATCAGCATCAGCTGTAACAGTTTATGAAAATGAAGGTACAAAAGTTGATTTTGATGGTCAATTGCGTCTTCTTTTAGAAAAACAAGCCTCAAAAGTGAAAGGTCAATCTTCAACAAGTGGTCACACTGACTTAAAGAATAATGGTTCTCGTTTCGGTATTTCTATCAAACATAATATCAATGAGAATCTCTACGGTTTTGGTCGTCATGAGACTCGCCTTGACAGTAATTCTAAAAATGCTGCAGGATGGGGCGATGTTAAAACAAAATATGCTTACGTCGGTTTAGGTGGCTATGGTCATGAAATTTCTTTTGGTAAACAAGTTGTAATCGGTGATAGCATTGGTCAAGCTGGTTTTGATAAAGTATACGATGTTGGTAGTACTGGAATTAGAATTAAATATCCAGCAAACTCAGCAGACAAAAAAGGTTTTGATATCCTTACTTCAGATTCTGATTCAGCAATTAACTATACCTATACAGGCATTGAAGGTTTGACGTTAGGTGCTAACTATAATGTTGCAAATGAGCGTGATAAGAAGACGGGAGAAGTAAAAGTAGATTCTGCTAAATCTGGCTTTGGTTTAGGTGCTAAATACACAGCTAAGATTGCGGAAAGTCAATCTGTAACTGTGGCAGCAGGTTATACTCATGATGACTATAAATCTGGAGCTGTTAATAAGAAAGACAAAGATGGTGTATACTTTGGTCTTAAATATGTCAATGCTCCATTTACTGTAGCTGTTGACGGTGGTCATGGTGTTGTAAAAACAGATAATGTTAAAGAGAAAATTGACTTCGTAAGAACTGGTGCAAGATTTGATGTTACTCCAAAATCTGGCGTGTATGGAAACTACTCTTATGGTACTTACAAAGATAAAGCTTACAAAGCAACAGCTCATCAATTCATGTTAGGTGCAGACTATAAATTACATAAACAAGTTGTTACCTTTGTTGAAGGTCGTTTAATCAAGAACAAAGACAGTAATAACAAAAAAGTTACTGACAAAGCACTTGGTGTTGGTTTACGTGTATTATGGTAA

>gi|284929974|gb|GU323689.1| Haemophilus parasuis strain Hs-DY03 outer membrane protein P2 precursor (ompP2) gene, complete cds

ATGAAAAAAACACTAGTAGCATTAGCAGTAGCGGCATTTGCAGCATCAGCATCAGCTGTAACAGTTTATGAAAATGAAGGTACAAAAGTTGATTTTGATGGTCAATTGCGTCTTCTTTTAGAAGAACAAGCCACAAAAGAGAAAGGTCAATCTTCAACACGTGGTCACACTAACTTAAAGAATAATAGTTCTCGTTTCGGTATTTCTATCAAACATAATATCAATGAGAATCTCTACGGTTTTGGTCGTTATGAGACTCGCCTTGACAGTAATTCTGAAAATGCTGCAGGATGGGGCGATGTTAAAACAAAATATGCTTACGTCGGTTTAGGTGGCTATGGTCATGAAATTTCTTTTGGTAAACAAGCTGTAATCGGTGATAGCATTGGTCAAGCTGGTTTTGATAAAGTATACGGTGTTGGTACTGGTGGAATTAAATATTCAGCAAACAACACAAACAAAAAAGGTTTTGATATCCTTACTTCAGATTCTGATTCAGCAATTAACTATACCTATACAGGCATTGAAGGTTTGACGTTAGGTGCTAACTATAATGTTGCAAATGAGCGTGATAAGAAGACGGGAGAAGTAAATGTAGGTTCTACTAAATCTGGCTTTGGTTTAGGTGCTAAATACACAGCTAAGATTGCGGAAAGTCAATCTGTAACTGTGGCAGCAGGTTATACTCATGATGACTATAAATCTGGATCTGTTAATAAGAAAGACAAAGATGGTGTATACTTTGGTCTTAAATATGTCAACTCTCCATTTACTGTAGCTGTTGATGGTGGTCATGGTGTTGAAAAAACAGGTAATGTTAAAGAGAAAATTGACTTCGTAAGAACTGGCGCAAGATTTGATGTTACTCCAAAATCTGGCGTGTATGGAAACTACTCTTATGGTACTTACAAAGATAAAGCTTACAAAGCAACAGCTCATCAATTCATGTTAGGTGCAGACTATAAATTACATAAACAAGTTGTTACCTTTGTTGAAGGTCGTTTAATCAAGAACAAAGACAGTAATAACAAAAAAGTTACTGACCAAGCACTTGGTGTTGGTTTACGTGTATTATGGTAA

>gi|284929976|gb|GU323690.1| Haemophilus parasuis strain Hs-DY04 outer membrane protein P2 precursor (ompP2) gene, complete cds

ATGAAAAAAACACTAGTAGCATTAGCAGTAGCGGCATTTGCAGCATCAGCATCAGCTGTAACAGTTTATGAAAATGAAGGTACAAAAGTTGATTTTGATGGTCAATTGCGTCTTCTTTTAGAAGAACAAGCCACAAAAGAGAAAGGTCAATCTTCAACAGGTGGTCACACTAACTTAAAGAATAATGGTTCTCGTTTCGGTATTTCTATCAAACATAATATCAATGAGAATCTCTACGGTTTTGGTCGTTATGAGACTCGCCTTGACAGTAATTCTAAAAATGCTGCAGGATGGGGCGATGTTAAAACAAAATATGCTTACGTCGGTTTAGGTGGCTATGGTCATGAAATTTCTTTTGGTAAACAAGATGTAATCGGTGATAGCATTGGTCAAGCTGGTTTTGATAAAGTATACGGTGTTGGTACTGGTGGAATTAAATATTCAGCAAACAACACAAACAAAAAAGGTTTTGATATCCTTACTTCAGATTCTGATTCAGCAATTAACTATACCTATACAGGCATTGAAGGTTTGACGTTAGGTGCTAACTATAATGTTGCAAATGAGCGTGATAAGAAGACGGGAGAAGTAAATGTAGGTTCTACTAAATCTGGCTTTGGTTTAGGTGCTAAATACACAGCTAAGATTGCGGAAAGTCAATCTGTAACTGTGGCAGCAGGTTATACTCATGATGACTATAAATCTGGATCTGTTAATAAGAAAGACAAAGATGGTGTATACTTTGGTCTTAAATATGTCAACGCTCCATTTACTGTAGCTGTTGATGGTGGTCATGGTGTTGAAAAAACAGGTAATGTTAAAGAGAAAATTGACTTCGTAAGAACTGGCGCAAGATTTGATGTTACTCCAAAATCTGGCGTGTATGGAAACTACTCTTATGGTACTTACAAAGATAAAGCTTACAAAGCAACAGCTCATCAATTCATGTTAGGTGCAGACTATAAATTACATAAACAAGTTGTTACCTTTGTTGAAGGTCGTTTAATCAAGAACAAAGACAGTAATAACAAAAAAGTTACTGACCAAGCACTTGGTGTTGGTTTACGTGTATTATGGTAA

>gi|284929978|gb|GU323691.1| Haemophilus parasuis strain Hs-DY05 outer membrane protein P2 precursor (ompP2) gene, complete cds

ATGAAAAAAACACTAGTAGCATTAGCAGTAGCGGCATTTGCAGCATCAGCATCAGCTGTAACAGTTTATGAAAATGAAGGTACAAAAGTTGATTTTGATGGTCAATTGCGTCTTCTTTTAGAAGAACAAGCCACAAAAGAGAAAGGTCAATCTTCAACAGGTGGTCACACTAACTTAAAGAATAATGGTTCTCGTTTCGGTATTTCTATCAAACATAATATCAATGAGAATCTCAACGGTTTTGGTCGTTATGAGACTCGCCTTGACAGTAATTCTAAAAATGCTGCAGGATGGGGCGATGTTAAAACAAAATATGCTTACGTCGGTTTAGGTGGCTATGGTCATGAAATTTCTTTTGGTAAACAAGATGTAATCGGTGATAGCATTGGTCAAGCTGGTTTTGATAAAGTATACGATGTTGGTACTGGTGGAATTAAATATTCAGCAAACAACACAAACAAAAAAGGTTTTGATATCCTTACTTCAGATTCTGATTCAGCAATTAACTATACCTATAAAGGCATTGAAGGTTTGACGTTAGGTGCTAACTATAATGTTGCAAATGAGCGTGATAAGAAGACGGGAGAAGTAAATGTAGGTTCTACTAAATCTGGCTTTGGTTTAGGTGCTAAATACACAGCTAAGATTGCGGAAAGTCAATCCGTAACTGTGGCAGCAGGTTATACTCATGATGACTATAAATCTGGATCTGTTAATAAGAAAGACAAAGATGGTGTATACTTTGGTCTTAAATATGTCAACGCTCCATTTACTGTAGCTGTTGATGGTGGTCATGGTGTTGAAAAAACAGGTAATGTTAAAGAGAAAATTGACTTCGTAAGAACTGGCGCAAGATTTGATGTTACTCCAAAATCTGGCGTGTATGGAAACTACTCTTATGGTACTTACAAAGATAAAGCTTACAAAGCAACAGCTCATCAATTCATGTTAGGTGCAGACTATAAATTACATAAACAAGTTGTTACCTTTGTTGAAGGTCGTTTAATCAAGAACAAAGACAGTAATAACAAAAAAGTTACTGACCAAGCACTTGGTGTTGGTTTACGTGTATTATGGTAA

>gi|284929980|gb|GU323692.1| Haemophilus parasuis strain Hs-DY06 outer membrane protein P2 precursor (ompP2) gene, complete cds

ATGAAAAAAACACTAGTAGCGTTAGCAGTAGCGGCATTTGCAGCATCAGCATCAGCTGTAACAGTTTATGAAAATGAAGGTACAAAAGTTGATTTTGATGGTCAATTGCGTCTTCTTTTAGAAAAACAAGCCTCAAAAGTGAAAGGTCAATCTTCAACAGATGGTCACACTAACTTAAAGAATAATAGTTCTCGTTTCGGTATTTCTATTAAACATAATATCAATGAGAATCTCTACGGTTTTGGTCGTTATGAGACTCGCCTTGGCAGTGGTTCTAAAAATGCTGCAAAATGGGGTGATGTTACAACAGATGAGGCTTACGTTGGTTTAGGTGGCTATGGTCATGAAATTTCTTTTGGTAAACAAGCTGTAATCGGTGATAGCATTGGTCAAGCTGGTTTTGATAAAGTATACGGTGTTGGTACTGGTGGAATTAAATATACATATAAGGTAAATAAGCCTATCACTGTGAACAATCAACAGGGTACATTTAAATATTCAGCACCTCAAGAAGGTTTTGATATCCTTACTCAATCTTCTGATTCAGCAATTAACTATACCTATACAGGTATTGAAGGTTTGACGTTAGGTGCTAACTATAATGTTGCAAATGAGCGTGAGAAGGCAGATGTAAAAGTAGATTCTATTAAATCTGGCTTTGGTTTAGGTGCTAAATACACAGCTAAGATCGCAGAAAGTCAATCTGTAACTGTGGCAGCAGGTTATACTCATGATGACTACAAATCTGGATCTGTTAAACTAAAAGGTAAATTTGTTCAAACAAATGGTACATCTACAGACCATACCTATACAGAGTCATTTAATAAGAAAGACAAAGATGGTGTATACTTTGGTCTTAAATATGTCAATGCTCCATTTACTGTAGCTGTTGATGGTGGTCATGGTGTTGTAAAAACAGATGATGTCAAAGAGAAAATTAACTTCGTAAGAACTGGCGCAAGATTTGATGTTACTCCAAAATCTGGCGTGTATGGAAACTACTCTTATGGTACTTACAAAGTTGAAGATTACAAAGCAACAGCTCATCAATTCATGTTAGGTGCAGACTATAAATTACATAAACAAGTCGTTACCTTTGTTGAAGGTCGTTTAATCAAGAACAAAGACAGTGATAACAACAAAGTTACTGACAAAGCACTTGGTGTTGGTTTACGTGTATTATGGTAA

>gi|284929982|gb|GU323693.1| Haemophilus parasuis strain Hs-DY07 outer membrane protein P2 precursor (ompP2) gene, complete cds

ATGAAAAAAACACTAGTAGCGTTAGCAGTAGCGGCATTTGCAGCATCAGCATCAGCTGTAACAGTTTATGAAAATGAAGGTACAAAAGTTGATTTTGATGGTCAATTGCGTCTTCTTTTAGAAAAACAAGTGAAAGGTCAATCTTCAACAGATGGTCACACTAACTTAAAGAATAATAGTTCTCGTTTCGGTATTTCTATCAAACATAATATCAATGAGAATCTTTACGGTTTTGGTCGTTATGAGACTCGCCTTGGCAGTGGTTCTAAATATGCTGCAAAATGGGGTGATGTTACAACAGATGAGGCTTACGTTGGTTTAGGTGGCTATGGTCATGAAATTTCTTTTGGTAAACAAGCTGTAATCGGTGATAGCATTGGTCAAGCTGGTTTTGATAAAGTATACGGTGTTGGTACTGGTGGAATTAAATATACATATAAGGTAAATGAGTCTATCACTGTGGACAATAAACGGGGTACATTTAAATATTCAGCACCTCAAGAAGGTTTTGATATCCTTACTCAATCTTCTGATTCAGCAATTAACTATACCTATACAGGTATTGAAGGTTTGACGTTAGGTGCTAACTATAATGTTGCAAATGAGCGTGAGGTAGATTCTATTAAATCTGGCTTTGGTTTAGGTGCTAAATACACAGCTAAGATTGCAGAAAGTCAATCTGTAACTGTGGCAGCAGGTTATACTCATGATGACTACAAATCTGGATCTGTTAAACTAAAAGGTAAATTTGTTGAAGCAGGTGGTAAATC

TACAGACCATACCTATACAGAAAAACCATTTAATAAGAAAGACAAAGATGGTGTGTACTTTGGTCTTAAATATGTCAACGCTCCATTTACTGTAGCTGTTGATGGTGGTCATGGTGTTGTAAAAACAGATGATGTTAAAGAGAAAATTAACTTCGTAAGAACTGGCGCAAGATTTGATGTTACTCCAAAATCTGGCGTGTATGGAAACTACTCTTATGGTACTTATAAAGTTGAAGCTTTCAAAGCAACTGCTCATCAATTCATGTTAGGTGCAGACTATAAATTACATAAACAAGTTGTTACCTTTGTTGAAGGTCGTTTAATCAAGAACAAAGACAGTGATAACAACAAAGTTACTGACAAAGCACTTGGTGTTGGTTTACGTGTATTATGGTAA

>gi|284929984|gb|GU323694.1| Haemophilus parasuis strain Hs-DY08 outer membrane protein P2 precursor (ompP2) gene, complete cds

ATGAAAAAAACACTAGTAGCGTTAGCAGTAGCGACATTTGCAGCATCAGCATCAGCTGTAACAGTTTATGAAAATGAAGGTACAAAAGTTGATTTTGATGGTCAATTGCGTCTTCTTTTAGAAAAACAAGCCTCAAAAGAGAAAGGTAAATCTTCAACAGATGGTCACACTAACTTAAAGAATAATAGTTCTCGTTTCGGTATTTCTATCAAACATAATATCAATGAGAATCTCTACGGTTTTGGTCGTTATGAGACTCGCCTTGGCAGTGGTTCTAAAAATGCTGCAAAATGGGGTGATGTTACAACAGATGAGGCTTACGTTGGTTTAGGTGGCTATGGTCATGAAATTTCTTTTGGTAAACAAGCTGTAATCGGTGATAGCATTGGTCAAGCTGGTTTTGATAAAGTATACGGTGTTGGTACTGGTGGAATTAAATATACATATGAGGTAGAAGAGCCTATCACTGTGAACAATACACAGGGTACATCACAGGGTACATTTAAATATTCAGCACCTCAAGAAGGTTTTGATATCCTTACTCAATCTTCTGATTCAGCAATTAACTATACCTATACAGGTATTGAAGGTTTGACGTTAGGTGCTAACTATAATGTTGCAAATGAGCGTGAGAAGGCAGATGTAAAAGTAGATTCTATTAAATCTGGCTTTGGTTTAGGTGCTAAATACACAGCTAAGATTGCAGAAAGTCAATCTGTAACTGTGGCAGCAGGTTATACTCATGATGACTACAAATCTGGATCTGTTAAACTAAAAGGTAAATTTGTTGAAGCAGGTGGTAAATCTACAGACCATATCCATACAGGAAAACCATTTAATAAGAAAGACAAAGATGGTGTGTACTTTGGTCTTAAATATGTCAACGCTCCATTTACTGTAGCTGTTGATGGTGGTCATGGTGTTGTAAAAACAGATGATGTTAAAGAGAAAATTAACTTCGTAAGAACTGGCGCAAGATTTGATGTTACTCCAAAATCTGGCGTGTATGGAAACTACTCTTATGGTACTTATAAAGTTGAAGATTTCAAAGTAACTGCTCATCAATTCATGTTAGGTGCAGACTATAAATTACATAAACAAGTTGTTACCTTTGTTGAAGGTCGTTTAATCAAGAACAAAGACAGTGATAACAACAAAGTTACTGACAAAGCACTTGGTGTTGGTTTACGTGTATTATGGTAA

>gi|284929986|gb|GU323695.1| Haemophilus parasuis strain Hs-DY09 outer membrane protein P2 precursor (ompP2) gene, complete cds

ATGAAAAAAACACTAGTAGCATTAGCAGTAGCGGCATTTGCAGCATCAGCGTCAGCTGTAACAGTTTATGAAAATGAAGGTACAAAAGTTGATTTTGATGGTCAATTGCGTCTTCTTTTAGAAGAACAAGCCACAAAAGAGAAAGGTCAATCTTCAACAGGTGGTCACACTAACTTAAAGAATAATGGTTCTCGTTTCGGTATTTCTATCAAACATAATATCAATGAGAATCTCTACGGTTTTGGTCGTTATGAGACTCGCCTTGGCCGTAATTCTAAAAATGATGCAGGATGGGGGGATGTTACAACAGAAAAGGCTTACGTCGGTTTAGGCGGCTATGGTCATGAAATTTCTTTTGGTAAACAAGCTGTAATCGGTGATAGCATTTGTCAAGCTGGTTTTGATAAAGTATACGGTGTTGGTACTGGTGGAATTAAATATTCAGCAAACAACACAAACAAAAAAGGTTTTGATATCCTTACTGCATCTCCTGATTCAGCAATTAACTATACCTATACAGGCATTGAAGGTTTGACGTTAGGTGCTAACTATAATGTTGCAAATGAGCGTGATAATAAGGGAGAAGTAAAGGTAGATTCTACTAAATCTGGCTTTGGTTTAGGTGCTAAATACACAGCTAAGATTGCGGAAAGTCAATCTGTAACTGTGGCAGCAGGTTATACTCATGATAACTATAAATCTGGAGCTGTTAATAAGAAAGACAAAGATGGTGTATACTTTGGTCTTAAATATGTCAATGCTCCATTTACTGTAGCTGTTGATGGTGGTCATGGTGTTGTAAAAACAGATAATGTTAAAGAGAAAATTGACTTCGTAAGAACTGGTGCAAGATTTGATGTTACTCCAAAATCTGGCGTGTATGGAAACTACTCTTATGGTACTTACAAAGATAAAGCTTACAAAGCAACAGCTCATCAATTCATGTTAGGTGCAGACTATAAATTACATAAACAAGTTGTTACCTTTGTTGAAGGTCGTTTAATCAAGAACAAAGACAGTAATAACAAAAAAGTTACTGACCAAGCACTTGGTGTTGGTTTACGTGTATTATGGTAA

>gi|284929988|gb|GU323696.1| Haemophilus parasuis strain Hs-DY10 outer membrane protein P2 precursor (ompP2) gene, complete cds

ATGAAAAAAACACTAGTAGCATTAGCAGTAGCGGCATTTGCAGCATCAGCATCAGCTGTAACAGTTTATGAAAATGAAGGTACAAAAGTTGATTTTGATGGTCAATTGCGTCTTCTTTTAGAAGAACAAGCCACAAAAGAGAAAGGTCAATCTTCAACACGTGGTCACACTAACTTAAAGAATAATAGTTCTCGTTTCGGTATTTCTATCAAACATAATATCAATGAGAATCTCTACGGTTTTGGTCGTTATGAGACTCGCCTTGACAGTAATTCTGAAAATGCTGCAGGATGGGGCGATGTTAAAACAAAATATGCTTACGTCGGTTTAGGTGGCTATGGTCATGAAATTTCTTTTGGTAAACAAGCTGTAATCGGTGATAGCATTGGTCAAGCTGGTTTTGATAAAGTATACGGTGTTGGTACTGGTGGAATTAAATATTCAGCAAACAACACAAACAAAAAAGGTTTTGATATCCTTACTTCAGATTCTGATTCAGCAATTAACTATACCTATACAGGCATTGAAGGTTTGACGTTAGGTGCTAACTATAATGTTGCAAATGAGCGTGATAAGAAGACGGGAGAAGTAAATGTAGGTTCTACTAAATCTGGCTTTGGTTTAGGTGCTAAATACACAGCTAAGATTGCGGAAAGTCAATCTGTAACTGTGGCAGCAGGTTATACTCATGATGACTATAAATCTGGATCTGTTAATAAGAAAGACAAAGATGGTGTATACTTTGGTCTTAAATATGTCAACTCTCCATTTACTGTAGCTGTTGATGGTGGTCATGGTGTTGAAAAAACAGGTAATGTTAAAGAGAAAATTGACTTCGTAAGAACTGGCGCAAGATTTGATGTTACTCCAAAATCTGGCGTGTATGGAAACTACTCTTATGGTACTTACAAAGATAAAGCTTACAAAGCAACAGCTCATCAATTCATGTTAGGTGCAGACTATAAATTACATAAACAAGTTGTTACCTTTGTTGAAGGTCGTTTAATCAAGAACAAAGACAGTAATAACAAAAAAGTTACTGACCAAGCACTTGGTGTTGGTTTACGTGTATTATGGTAA

>gi|284929990|gb|GU323697.1| Haemophilus parasuis strain Hs-DY11 outer membrane protein P2 precursor (ompP2) gene, complete cds

ATGAAAAAAACACTAGTAGCGTTAGCAGTAGCGGCATTTGCAGCATCAGCATCAGCTGTAACAGTTTATGAAAATGAAGGTACAAAAGTTGATTTTGATGGTCAATTGCGTCTTCTTTTAGAAAAACAAGTGAAAGGTCAATCTTCAACAGATGGTCACACTAACTTAAAGAATAATAGTTCTCGTTTCGGTATTTCTATCAAACATAATATCAATGAGAATCTTTACGGTTTTGGTCGTTATGAGACTCGCCTTGGCAGTGGTTCTAAAAATGCTGCAAAATGGGGTGATGTTACAACAGATGAGGCTTACGTTGGTTTAGGTGGCTATGGTCATGAAATTTCTTTTGGTAAACAAGCTGTAATCGGTGATAGCATTGGTCAAGCTGGTTTTGATAAAGTATACGGTGTTGGTACTGGTGGAATTAAATATACATATAAGGTAAATGAGTCTATCACTGTGGACAATAAACGGGGTACATTTAAATATTCAGCACCTCAAGAAGGTTTTGATATCCTTACTCAATCTTCTGATTCAGCAATTAACTATACCTATACAGGTATTGAAGGTTTGACGTTAGGTGCTAACTATAATGTTGCAAATGAGCGTGAGGTAGATTCTATTAAATCTGGCTTTGGTTTAGGTGCTAAATACACAGCTAAGATTGCAGAAAGTCGATCTGTAACTGTGGCAGCAGGTTATACTCATGATGACTACAAATCTGGATCTGTTAAACTAAAAGGTAAATTTGTTGAAGCAGGTGGTAAATCTACAGACCATACCTATACAGAAAAACCATTTAATAAGAAAGACAAAGATGGTGTGTACTTTGGTCTTAAATATGTCAACGCTCCATTTACTGTAGCTGTTGATGGTGGTCATGGTGTTGTAAAAACAGATGATGTTAAAGAGAAAATTAACTTCGTAAGAACTGGCGCAAGATTTGATGTTACTCCAAAATCTGGCGTGTATGGAAACTACTCTTATGGTACTTATAAAGTTGAAGCTTTCAAAGCAACTGCTCATCAATTCATGTTAGGTGCAGACTATAAATTACATAAACAAGTTGTTACCTTTGTTGAAGGTCGTTTAATCAAGAACAAAGACAGTGATAACAACAAAGTTACTGACAAAGCACTTGGTGTTGGTTTACGTGTATTATGGTAA

>gi|284929992|gb|GU323698.1| Haemophilus parasuis strain Hs-DY12 outer membrane protein P2 precursor (ompP2) gene, complete cds

ATGAAAAAAACACTAGTAGCATTAGCAGTAGCGGCATTTGCAGCATCAGCATCAGCTGTAACAGTTTATGAAAATGAAGGTACAAAAGTTGATTTTGATGGTCAATTGCGTCTTCTTTTAGAAGAACAAGCCACAAAAGAGAAAGGTCAATCTTCAACAGGTGGTCACACTAACTTAAAGAATAATGGTTCTCGTTTCGGTATTTCTATCAAACATAATATCAATGAGAATCTCTACGGTTTTGGTCGTTATGAGACTCGCCTTGGCCGTAATTCTAAAAATGATGCAGGATGGGGGGATGTTACAACAGAAAAGGCTTACGTCGGTTTAGGCGGCTATGGTCATGAAATTTCTTTTGGTAAACAAGCTGTAATCGGTGATAGCATTGGTCAAGCTGGTTTTGATAAAGTATACGGTGTTGGTACTGGTGGAATTAAATATTCAGCAAACAACACAAACAAAAAAGGTTTTGATATCCTTACTGCATCTTCTGATTCAGCAATTAACTATACCTATACAGGCATTGAAGGTTTGACGTTAGGTGCTAACTATAATGTTGCAAATGAGCGTGATAATAAGGGAGAAGTAAAGGTAGATTCTACTAAATCTGGCTTTGGTTTAGGTGCTAAATACACAGCTAAGATTGCGGAAAGTCAATCTGTAACTGTGGCAGCAGGTTATACTCATGATGACTATAAATCTGGAGCTGTTAATAAGAAAGACAAAGATGGTGTATACTTTGGTCTTAAATATGTCAATGCTCCATTTACTGTAGCTGTTGATGGTGGTCATGGTGTTGTAAAAACAGATAATGTTAAAGAGAAAATTGACTTCGTAAGAACTGGTGCAAGATTTGATGTTACTCCAAAATCTGGCGTGTATGGAAACTACTCTTATGGTACTTACAAAGATAAAGCTTACAAAGCAACAGCTCATCAATTCATGTTAGGTGCAGACTATAAATTACATAAACAAGTTGTTACCTTTGTTGAAGGTCGTTTAATCAAGAACAAAGACAGTAATAACAAAAAAGTTACTGACCAAGCACTTGGTGTTGGTTTACGTGTATTATGGTAA

>gi|284929994|gb|GU323699.1| Haemophilus parasuis strain Hs-DY13 outer membrane protein P2 precursor (ompP2) gene, complete cds

ATGAAAAAAACACTAGTAGCATTAGCAGTAGCGGCATTTGCAGCATCAGCATCAGCTGTAACAGTTTATGAAAATGAAGGTACAAAAGTTGATTTTGATGGTCAATTGCGTCTTCTTTTAGAAGAACAAGCCACAAAAGAGAAAGGTCAATCTTCAACACGTGGTCACACTAACTTAAAGAATAATAGTTCTCGTTTCGGTATTTCTATCAAACATAATATCAATGAGAATCTCTACGGTTTTGGTCGTTATGAGACTCGCCTTGACAGTAATTCTGAAAATGCTGCAGGATGGGGCGATGTTAAAACAAAATATGCTTACGTCGGTTTAGGTGGCTATGGTCATGAAATTTCTTTTGGTAAACAAGCTGTAATCGGTGATAGCATTGGTCAAGCTGGTTTTGATAAAGTATACGGTGTTGGTACTGGTGGAATTAAATATTCAGCAAACAACACAAACAAAAAAGGTTTTGATATCCTTACTTCAGATTCTGATTCAGCAATTAACTATACCTATACAGGCATTGAAGGTTTGACGTTAGGTGCTAACTATAATGTTGCAAATGAGCGTGATAAGAAGACGGGAGAAGTAAATGTAGGTTCTACTAAATCTGGCTTTGGTTTAGGTGCTAAATACACAGCTAAGATTGCGGAAAGTCAATCTGTAACTGTGGCAGCAGGTTATACTCATGATGACTATAAATCTGGATCTGTTAATAAGAAAGACAAAGATGGTGTATACTTTGGTCTTAAATATGTCAACTCTCCATTTACTGTAGCTGTTGATGGTGGTCATGGTGTTGAAAAAACAGGTAATGTTAAAGAGAAAATTGACTTCGTAAGAACTGGCGCAAGATTTGATGTTACTCCAAAATCTGGCGTGTATGGAAACTACTCTTATGGTACTTACAAAGATAAAGCTTACAAAGCAACAGCTCATCAATTCATGTTAGGTGCAGACTATAAATTACATAAACAAGTTGTTACCTTTGTTGAAGGTCGTTTAATCAAGAACAAAGACAGTAATAACAAAAAAGTTACTGACCAAGCACTTGGTGTTGGTTTACGTGTATTATGGTAA

>gi|284929996|gb|GU323700.1| Haemophilus parasuis strain Hs-DY14 outer membrane protein P2 precursor (ompP2) gene, complete cds

ATGAAAAAAACACTAGTAGCATTAGCAGTAGCGGCATTTGCAGCATCAGCATCAGCTGTAACAGTTTATGAAAATGAAGGTACAAAAGTTGATTTTGATGGTCAATTGCGTCTTCTTTTAGAAGAACAAGCCACAAAAGAGAAAGGTCAATCTTCAACAGGTGGTCACACTAACTTAAAGAATAATGGTTCTCGTTTCGGTATTTCTATCAAACATAATATCAATGAGAATCTCTACGGTTTTGGTCGTTATGAGACTCGCCTTGACAGTAATTCTAAAAATGCTGCAGGATGGGGCGATGTTAAAACAAAATATGCTTACGTCGGTTTAGGTGGCTATGGTCATGAAATTTCTTTTGGTAAACAAGCTGTAATCGGTGATAGCATTGGTCAAGCTGGTTTTGATAAAGTATACGGTGTTGGTACTGGTGGAATTAAATATTCAGCAAACAACACAAACAAAAAAGGTTTTGATATCCTTACTTCAGATTCTGATTCAGCAATTAACTATACCTATACAGGCATTGAAGGTTTGACGTTAGGTGCTAACTATAATGTTGCAAATGAGCGTGATAAGAAGACGGGAGAAGTAAATGTAGGTTCTACTAAATCTGGCTTTGGTTTAGGTGCTAAATACACAGCTAAGATTGCGGAAAGTCAATCTGTAACTGTGGCAGCAGGTTATACTCATGATGACTATAAATCTGGATCTGTTAATAAGAAAGACAAAGATGGTGTATACTTTGGTCTTAAATATGTCAACGCTCCATTTACTGTAGCTGTTGATGGTGGTCATGGTGTTGAAAAAACAGGTGATGTTAAAGAGAAAATTGACTTCGTAAGAACTGGCGCAAGATTTGATGTTACTCCAAAATCTGGCGTGTATGGAAACTACTCTTATGGTACTTACAAAGATAAAGCTTACAAAGCAACAGCTCATCAATTCATGTTAGGTGCAGACTATAAATTACATAAACAAGTTGTTACCTTTGTTGAAGGTCGTTTAATCAAGAACAAAGACAGTAATAACAAAAAAGTTACTGACCAAGCACTTGGTGTTGGTTTACGTGTATTATGGTAA

>gi|284929998|gb|GU323701.1| Haemophilus parasuis strain Hs-DY15 outer membrane protein P2 precursor (ompP2) gene, complete cds

ATGAAAAAAACACTAGTAGCATTAGCAGTAGCGGCATTTGCAGCATCAGCATCAGCTGTAACAGTTTATGAAAATGAAGGTACAAAAGTTGATTTTGATGGTCAATTGCGTCTTCTTTTAGAAGAACAAGCCACAAAAGAGAAAGGTCAATCTTCAACACGTGGTCACACTAACTTAAAGAATAATAGTTCTCGTTTCGGTATTTCTATCAAACATAATATCAATGAGAATCTCTACGGTTTTGGTCGTTATGAGACTCGCCTTGACAGTAATTCTGAAAATGCTGCAGGATGGGGCGATGTTAAAACAAAATATGCTTACGTCGGTTTAGGTGGCTATGGTCATGAAATTTCTTTTGGTAAACAAGCTGTAATCGGTGATAGCATTGGTCAAGCTGGTTTTGATAAAGTATACGGTGTTGGTACTGGTGGAATTAAATATTCAGCAAACAACACAAACAAAAAAGGTTTTGATATCCTTACTGACTCTTCTGATTCAGCAATTAACTATACCTATACAGGCATTGAAGGTTTGACGTTAGGTGCTAACTATAATGTTGCAAATGAGCGTGATAAGAAGACGGGAGAAGTAAATGTAGGTTCTACTAAATCTGGCTTTGGTTTAGGTGCTAAATACACAGCTAAGATTGCGGAAAGTCAATCTGTAACTGTGGCAGCAGGTTATACTCATGATGACTATAAATCTGGAGCTGTTAATAAGAAAGACAAAGATGGTGTATACTTTGGTCTTAAATATGTCAATGCTCCATTTACTGTAGCTGTTGATGGTGGTCATGGTGTTGTAAAAACAGATAATGTTAAAGAGAAAATTGACTTCGTAAGAACTGGCGCAAGATTTGATGTTACTCCAAAATCTGGCGTGTATGGAAACTACTCTTATGGTACTTACAAAGATAAAGCTTACAAAGCAACAGCTCATCAATTCATGTTAGGTGCAGACTATAAATTACATAAACAAGTTGTTACCTTTGTTGAAGGTCGTTTAATCAAGAACAAAGACAGTAATAACAAAAAAGTTACTGACCAAGCACTTGGTGTTGGTTTACGTGTATTATGGTAA

>gi|209968889|gb|EU852097.1| Haemophilus parasuis strain SW124 outer membrane protein P2 (ompP2) gene, complete cds

ATGAAAAAAACACTAGTAGCGTTAGCAGTAGCGGCATTTGCAGCATCAGCATCAGCTGTAACAGTTTATGAAAATGAAGGTACAAAAGTTGATTTTGATGGTCAATTGCGTCTTCTTTTAGAAAAACAAGCCTCAAAAGTGAAAGGTCAATCTTCAACAGATGGTCACACTAACTTAAAGAATAATAGTTCTCGTTTCGGTATTTCTATCAAACATAATATCAATGAGAATCTTTACGGTTTTGGTCGTTATGAGACTCGCCTTGACAGTGGTTCTAAAAATGCTGCAAAATGGGGTGATGTTACAACAGATGAGGCTTACGTTGGTTTAGGTGGCTATGGTCATGAAATTTCTTTTGGTAAACAAGCTGTAATCGGTGATAGCATTGGTCAAGCTGGTTTTGATAAAGTATACGGTGTTGGTACTGGTGGAATTAAATATACATATAAGGTAGATAAGCCTATCACTGGGAACAATAGACAGGGTACATTATATTCAGCACCTCAAAAAGGTTTTGATATCCTTACTCAATCTTCTGATTCAGCAATTAACTATACCTATACAGGTATTGAAGGTTTGACGTTAGGTGCTAACTATAATGTTGCAAATGAGCGTGAGAAGGCAGATGTAAAAGTAGATTCTATTAAATCTGGCTTTGGTTTAGGTGCTAAATACACAGCTAAGATTGCAGAAAGTCAATCTGTAACTGTGGCAGCAGGTTATACTCATGATGACTATAAATCTGGATCTGTTGAACTAAAAGGTAAATTTGTTCAAACAAATGGTACATCTACAAACCATACCTATACAGAGTCATTTAATAAGAAAGACAAAGATGGTGTATACTTTGGTCTTAAATATGTCAATGCTCCATTTACTGTAGCTGTTGATGGTGGTCATGGTGTTGTAAAAACAGATGATGTTAAAGAGAAAATTAACTTCGTAAGAACTGGCGCAAGATTTGATGTTACTCCAAAATCTGGCGTGTATGGAAACTACTCTTATGGTACTTACAAAGTTGAAGATTTCAAAGCAACTGCTCATCAATTCATGTTAGGTGCAGACTATAAATTACATAAACAAGTTGTTACCTTTGTTGAAGGTCGTTTAATCAAGAACAAAAACAGTGATAACAACAAAGTTACTGACAAAGCACTTGGTGTTGGTTTACGTGTATTATGGTAA

>gi|209968891|gb|EU852098.1| Haemophilus parasuis strain 131 outer membrane protein P2 (ompP2) gene, complete cds

ATGAAAAAAACACTAGTAGCGTTAGCAGTAGCGACATTTGCAGCATCAGCATCAGCTGTAACAGTTTATGAAAATGAAGGTACAAAAGTTGATTTTGATGGTCAGTTGCGTCTTCTTTTAGAAAAAAAAGCCTCAAAAGAGAAAGGTAAATCTTCAACAGATGGTCACACTAACTTAAAGAATAATAGTTCTCGTTTCGGTATTTCTATCAAACATAATATCAATGAGAATCTCTACGGTTTTGGTCGTTATGAGACTCGCCTTGGCAGTGGTTCTAAAAATGCTGCAAAATGGGGTGATGTTACAACAGATGAGGCTTACGTTGGTTTAGGTGGCTATGGTCATGAAATTTCTTTTGGTAAACAAGCTGTAATCGGTGATAGCATTGGTCAAGCTGGTTTTGATAAAGTATACGGTGTTGGTACTGGTGGAATTAAATATACATATAAGGTAGATGAGTCTATCACTGTGAACAATACACAGGGTACATTTAAATATTCAGCACCTCAAGAAGGTTTTGATATCCTTACTCAATCTTCTGATTCAGCAATTAACTATACCTATACAGGTATTGAAGGTTTGACGTTAGGTGCTAACTATAATGTTGCAAATGAGCGTGAGAAGGCAGATGTAAAAGTAGATTCTATTAAATCTGGCTTTGGTTTAGGTGCTAAATACACAGCTAAGATTGCAGAAAGTCAATCTGTAACTGTGGCAGCAGGTTATACTCATGATGACTACAAATCTGGATCTGTTCAACTAAAAGGTAAATTTGTTCAAGCAAATGGTACATCTACAGACCATACCTATACAGAGTCATTTAATAAGAAAAACAAAGATGGTGTATACTTTGGTCTTAAATATGTCAACGCTCCATTTACTGTAGCTGTTGATGGTGGTCATGGTGTTGTAAAAACAGATGATGTTAAAGAGAAAATTAACTTCGTAAGAACTGGCGCAAGATTTGATGTTACTCCAAAATCTGGCGTGTATGGAAACTACTCTTATGGTACTTACAAAGTTGAAGATTACAAAGCAACAGCTCATCAATTCATGTTAGGTGCAGACTATAAATTACATAAACAAGTTGTTACCTTTGTTGAAGGTCGTTTAATCAAGAACAAAGACAGTGATAACAACAAAGTTACTGACAAAGCACTTGGTGTTGGTTTACGTGTATTATGGTAA

>gi|209968893|gb|EU852099.1| Haemophilus parasuis strain C5 outer membrane protein P2 (ompP2) gene, complete cds

ATGAAAAAAACACTAGTAGCGTTAGCAGTAGCGACATTTGCAGCATCAGCATCAGCTGTAACAGTTTATGAAAATGAAGGTACAAAAGTTGATTTTGATGGTCAATTGCGTCTTCTTTTAGAAAAACAAGCCTCAAAAGAGAAAGGTAAATCTTCAACAGATGGTCACACTAACTTAAAGAATAATAGTTCTCGTTTCGGTATTTCTATCAAACATAATATCAATGAGAATCTCTACGGTTTTGGTCGTTATGAGACTCGCCTTGGCAGTGGTTCTAAAAATGCTGCAAAATGGGGTGATGTTACAACAGATGAGGCTTACGTTGGTTTAGGTGGCTATGGTCATGAAATTTCTTTTGGTAAACAAGCTGTAATCGGTGATAGCATTGGTCAAGCTGGTTTTGATAAAGTATACGGTGTTGGTACTGGTGGAATTAAATATACATATGAGGTAGAAGAGCCTATCACTGTGAACAATACACAGGGTACATCACAGGGTACATTTAAATATTCAGCACCTCAAGAAGGTTTTGATATCCTTACTCAATCTTCTGATTCAGCAATTAACTATACCTATACAGGTATTGAAGGTTTGACGTTAGGTGCTAACTATAATGTTGCAAATGAGCGTGAGAAGGCAGATGTAAAAGTAGATTCTATTAAATCTGGCTTTGGTTTAGGTGCTAAATACACAGCTAAGATTGCAGAAAGTCAATCTGTAACTGTGGCAGCAGGTTATACTCATGATGACTACAAATCTGGATCTGTTAAACTAAAAGGTAAATTTGTTGAAGCAGGTGGTAAATCTACAGACCATATCCATACAGGAAAACCATTTAATAAGAAAGACAAAGATGGTGTGTACTTTGGTCTTAAATATGTCAACGCTCCATTTACTGTAGCTGTTGATGGTGGTCATGGTGTTGTAAAAACAGATGATGTTAAAGAGAAAATTAACTTCGTAAGAACTGGCGCAAGATTTGATGTTACTCCAAAATCTGGCGTGTATGGAAACTACTCTTATGGTACTTATAAAGTTGAAGATTTCAAAGTAACTGCTCATCAATTCATGTTAGGTGCAGACTATAAATTACATAAACAAGTTGTTACCTTTGTTGAAGGTCGTTTAATCAAGAACAAAGACAGTGATAACAACAAAGTTACTGACAAAGCACTTGGTGTTGGTTTACGTGTATTATGGTAA

>gi|209968859|gb|EU741915.1| Haemophilus parasuis strain 84-15995 outer membrane protein P2 (ompP2) gene, complete cds

ATGAAAAAAACACTAGTAGCATTAGCAGTAGCGGCATTTGCAGCATCAGCATCAGCTGTAACAGTTTATGAAAATGAAGGTACAAAAGTTGATTTTGATGGTCAATTGCGTCTTCTTTTAGAAGAACAAGCCACAAAAGAGAAAGGTCAATCTTCAACACGTGGTCACACTAACTTAAAGAATAATAGTTCTCGTTTCGGTATTTCTATCAAACATAATATCAATGAGAATCTCTACGGTTTTGGTCGTTATGAGACTCGCCTTGACAGTAATTCTGAAAATGCTGCAGGATGGGGCGATGTTAAAACAAAATATGCTTACGTCGGTTTAGGTGGCTATGGTCATGAAATTTCTTTTGGTAAACAAGCTGTAATCGGTGATAGCATTGGTCAAGCTGGTTTTGATAAAGTATACGGTGTTGGTACTGGTGGAATTAAATATTCAGCAAACAACACAAACAAAAAAGGTTTTGATATCCTTACTGACTCTTCTGATTCAGCAATTAACTATACCTATACAGGCATTGAAGGTTTGACGTTAGGTGCTAACTATAATGTTGCAAATGAGCGTGATAAGAAGACGGGAGAAGTAAATGTAGGTTCTACTAAATCTGGCTTTGGTTTAGGTGCTAAATACACAGCTAAGATTGCGGAAAGTCAATCTGTAACTGTGGCAGCAGGTTATACTCATGATGACTATAAATCTGGAGCTGTTAATAAGAAAGACAAAGATGGTGTATACTTTGGTCTTAAATATGTCAATGCTCCATTTACTGTAGCTGTTGATGGTGGTCATGGTGTTGTAAAAACAGATAATGTTAAAGAGAAAATTGACTTCGTAAGAACTGGCGCAAGATTTGATGTTACTCCAAAATCTGGCGTGTATGGAAACTACTCTTATGGTACTTACAAAGATAAAGCTTACAAAGCAACAGCTCATCAATTCATGTTAGGTGCAGACTATAAATTACATAAACAAGTTGTTACCTTTGTTGAAGGTCGTTTAATCAAGAACAAAGACAGTAATAACAAAAAAGTTACTGACCAAGCACTTGGTGTTGGTTTACGTGTATTATGGTAA

>gi|209968857|gb|EU741914.1| Haemophilus parasuis strain 84-22113 outer membrane protein P2 (ompP2) gene, complete cds

ATGAAAAAAACACTAGTAGCATTAGCAGTAGCGGCATTTGCAGCATCAGCATCAGCTGTAACAGTTTATGAAAATGAAGGTACAAAAGTTGATTTTGATGGTCAATTGCGTCTTCTTTTAGAAGAACAAGCCACAAAAGAGAAAGGTCAATCTTCAACAGGTGGTCACACTAACTTAAAGAATAATGGTTCTCGTTTCGGTATTTCTATCAAACATAATATCAATGAGAATCTCTACGGTTTTGGTCGTTATGAGACTCGCCTTGACAGTAATTCTAAAAATGCTGCAGGATGGGGCGATGTTAAAACAAAATATGCTTACGTCGGTTTAGGTGGCTATGGTCATGAAATTTCTTTTGGTAAACAAGCTGTAATCGGTGATAGCATTGGTCAAGCTGGTTTTGATAAAGTATACGGTGTTGGTACTGGTGGAATTAAATATTCAGCAAACAACACAAACAAAAAAGGTTTTGATATCCTTACTTCAGATTCTGATTCAGCAATTAACTATACCTATACAGGCATTGAAGGTTTGACGTTAGGTGCTAACTATAATGTTGCAAATGAGCGTGATAAGAAGACGGGAGAAGTAAATGTAGGTTCTACTAAATCTGGCTTTGGTTTAGGTGCTAAATACACAGCTAAGATTGCGGAAAGTCAATCTGTAACTGTGGCAGCAGGTTATACTCATGATGACTATAAATCTGGATCTGTTAATAAGAAAGACAAAGATGGTGTATACTTTGGTCTTAAATATGTCAACGCTCCATTTACTGTAGCTGTTGATGGTGGTCATGGTGTTGAAAAAACAGGTAATGTTAAAGAGAAAATTGACTTCGTAAGAACTGGCGCAAGATTTGATGTTACTCCAAAATCTGGCGTGTATGGAAACTACTCTTATGGTACTTACAAAGATAAAGCTTACAAAGCAACAGCTCATCAATTCATGTTAGGTGCAGACTATAAATTACATAAACAAGTTGTTACCTTTGTTGAAGGTCGTTTAATCAAGAACAAAGACAGTAATAACAAAAAAGTTACTGACCAAGCACTTGGTGTTGGTTTACGTGTATTATGGTAA

>gi|209968855|gb|EU741913.1| Haemophilus parasuis strain 84-17975 outer membrane protein P2 (ompP2) gene, complete cds

ATGAAAAAAACACTAGTAGCATTAGCAGTAGCGGCATTTGCAGCATCAGCATCAGCTGTAACAGTTTATGAAAATGAAGGTACAAAAGTTGATTTTGATGGTCAATTGCGTCTTCTTTTAGAAGAACAAGCCACAAAAGAGAAAGGTCAATCTTCAACACGTGGTCACACTAACTTAAAGAATAATAGTTCTCGTTTCGGTATTTCTATCAAACATAATATCAATGAGAATCTCTACGGTTTTGGTCGTTATGAGACTCGCCTTGACAGTAATTCTGAAAATGCTGCAGGATGGGGCGATGTTAAAACAAAATATGCTTACGTCGGTTTAGGTGGCTATGGTCATGAAATTTCTTTTGGTAAACAAGCTGTAATCGGTGATAGCATTGGTCAAGCTGGTTTTGATAAAGTATACGGTGTTGGTACTGGTGGAATTAAATATTCAGCAAACAACACAAACAAAAAAGGTTTTGATATCCTTACTTCAGATTCTGATTCAGCAATTAACTATACCTATACAGGCATTGAAGGTTTGACGTTAGGTGCTAACTATAATGTTGCAAATGAGCGTGATAAGAAGACGGGAGAAGTAAATGTAGGTTCTACTAAATCTGGCTTTGGTTTAGGTGCTAAATACACAGCTAAGATTGCGGAAAGTCAATCTGTAACTGTGGCAGCAGGTTATACTCATGATGACTATAAATCTGGATCTGTTAATAAGAAAGACAAAGATGGTGTATACTTTGGTCTTAAATATGTCAACGCTCCATTTACTGTAGCTGTTGATGGTGGTCATGGTGTTGAAAAAACAGGTAATGTTAAAGAGAAAATTGACTTCGTAAGAACTGGCGCAAGATTTGATGTTACTCCAAAATCTGGCGTGTATGGAAACTACTCTTATGGTACTTACAAAGATAAAGCTTACAAAGCAACAGCTCATCAATTCATGTTAGGTGCAGACTATAAATTACATAAACAAGTTGTTACCTTTGTTGAAGGTCGTTTAATCAAGAACAAAGACAGTAATAACAAAAAAGTTACTGACCAAGCACTTGGTGTTGGTTTACGTGTATTATGGTAA

>gi|209968853|gb|EU741912.1| Haemophilus parasuis strain H425 outer membrane protein P2 (ompP2) gene, complete cds

ATGAAAAAAACACTAGTAGCATTAGCAGTAGCGGCATTTGCAGCATCAGCATCAGCTGTAACAGTTTATGAAAATGAAGGTACAAAAGTTGATTTTGATGGTCAATTGCGTCTTCTTTTAGAAGAACAAGCCACAAAAGAGAAAGGTCAATCTTCAACAGGTGGTCACACTAACTTAAAGAATAATGGTTCTCGTTTCGGTATTTCTATCAAACATAATATCAATGAGAATCTCTACGGTTTTGGTCGTTATGAGACTCGCCTTGGCCGTAATTCTAAAAATGATGCAGGATGGGGGGATGTTACAACAGAAAAGGCTTACGTCGGTTTAGGCGGCTATGGTCATGAAATTTCTTTTGGTAAACAAGCTGTAATCGGTGATAGCATTGGTCAAGCTGGTTTTGATAAAGTATACGGTGTTGGTACTGGTGGAATTAAATATTCAGCAAACAACACAAACAAAAAAGGTTTTGATATCCTTACTGCATCTTCTGATTCAGCAATTAACTATACCTATACAGGCATTGAAGGTTTGACGTTAGGTGCTAACTATAATGTTGCAAATGAGCGTGATAATAAGGGAGAAGTAAAGGTAGATTCTACTAAATCTGGCTTTGGTTTAGGTGCTAAATACACAGCTAAGATTGCGGAAAGTCAATCTGTAACTGTGGCAGCAGGTTATACTCATGATGACTATAAATCTGGAGCTGTTAATAAGAAAGACAAAGATGGTGTATACTTTGGTCTTAAATATGTCAATGCTCCATTTACTGTAGCTGTTGATGGTGGTCATGGTGTTGTAAAAACAGATAATGTTAAAGAGAAAATTGACTTCGTAAGAACTGGTGCAAGATTTGATGTTACTCCAAAATCTGGCGTGTATGGAAACTACTCTTATGGTACTTACAAAGATAAAGCTTACAAAGCAACAGCTCATCAATTCATGTTAGGTGCAGACTATAAATTACATAAACAAGTTGTTACCTTTGTTGAAGGTCGTTTAATCAAGAACAAAGACAGTAATAACAAAAAAGTTACTGACCAAGCACTTGGTGTTGGTTTACGTGTATTATGGTAA

>gi|209968851|gb|EU741911.1| Haemophilus parasuis strain H465 outer membrane protein P2 (ompP2) gene, complete cds

ATGAAAAAAACACTAGTAGCGTTAGCAGTAGCGGCATTTGCAGCATCAGCATCAGCTGTAACAGTTTATGAAAATGAAGGTACAAAAGTTGATTTTGATGGTCAATTGCGTCTTCTTTTAGAAAAACAAGTGAAAGGTCAATCTTCAACAGATGGTCACACTAACTTAAAGAATAATAGTTCTCGTTTCGGTATTTCTATCAAACATAATATCAATGAGAATCTTTACGGTTTTGGTCGTTATGAGACTCGCCTTGGCAGTGGTTCTAAAAATGCTGCAAAATGGGGTGATGTTACAACAGATGAGGCTTACGTTGGTTTAGGTGGCTATGGTCATGAAATTTCTTTTGGTAAACAAGCTGTAATCGGTGATAGCATTGGTCAAGCTGGTTTTGATAAAGTATACGGTGTTGGTACTGGTGGAATTAAATATACATATAAGGTAAATGAGTCTATCACTGTGGACAATAAACGGGGTACATTTAAATATTCAGCACCTCAAGAAGGTTTTGATATCCTTACTCAATCTTCTGATTCAGCAATTAACTATACCTATACAGGTATTGAAGGTTTGACGTTAGGTGCTAACTATAATGTTGCAAATGAGCGTGAGGTAGATTCTATTAAATCTGGCTTTGGTTTAGGTGCTAAATACACAGCTAAGATTGCAGAAAGTCAATCTGTAACTGTGGCAGCAGGTTATACTCATGATGACTACAAATCTGGATCTGTTAAACTAAAAGGTAAATTTGTTGAAGCAGGTGGTAAATCTACAGACCATACCTATACAGAAAAACCATTTAATAAGAAAGACAAAGATGGTGTGTACTTTGGTCTTAAATATGTCAACGCTCCATTTACTGTAGCTGTTGATGGTGGTCATGGTGTTGTAAAAACAGATGATGTTAAAGAGAAAATTAACTTCGTAAGAACTGGCGCAAGATTTGATGTTACTCCAAAATCTGGCGTGTATGGAAACTACTCTTATGGTACTTATAAAGTTGAAGCTTTCAAAGCAACTGCTCATCAATTCATGTTAGGTGCAGACTATAAATTACATAAACAAGTTGTTACCTTTGTTGAAGGTCGTTTAATCAAGAACAAAGACAGTGATAACAACAAAGTTACTGACAAAGCACTTGGTGTTGGTTTACGTGTATTATGGTAA

>gi|209968849|gb|EU741910.1| Haemophilus parasuis strain H367 outer membrane protein P2 (ompP2) gene, complete cds

ATGAAAAAAACACTAGTAGCATTAGCAGTAGCGGCATTTGCAGCATCAGCATCAGCTGTAACAGTTTATGAAAATGAAGGTACAAAAGTTGATTTTGATGGTCAATTGCGTCTTCTTTTAGAAGAACAAGCCACAAAAGAGAAAGGTCAATCTTCAACAGGTGGTCACACTAACTTAAAGAATAATGGTTCTCGTTTCGGTATTTCTATCAAACATAATATCAATGAGAATCTCTACGGTTTTGGTCGTTATGAGACTCGCCTTGGCCGTAATTCTAAAAATGATGCAGGATGGGGGGATGTTACAACAGAAAAGGCTTACGTCGGTTTAGGCGGCTATGGTCATGAAATTTCTTTTGGTAAACAAGCTGTAATCGGTGATAGCATTGGTCAAGCTGGTTTTGATAAAGTATACGGTGTTGGTACTGGTGGAATTAAATATTCAGCAAACAACACAAACAAAAAAGGTTTTGATATCCTTACTGCATCTTCTGATTCAGCAATTAACTATACCTATACAGGCATTGAAGGTTTGACGTTAGGTGCTAACTATAATGTTGCAAATGAGCGTGATAATAAGGGAGAAGTAAAGGTAGATTCTACTAAATCTGGCTTTGGTTTAGGTGCTAAATACACAGCTAAGATTGCGGAAAGTCAATCTGTAACTGTGGCAGCAGGTTATACTCATGATGACTATAAATCTGGAGCTGTTAATAAGAAAGACAAAGATGGTGTATACTTTGGTCTTAAATATGTCAATGCTCCATTTACTGTAGCTGTTGATGGTGGTCATGGTGTTGTAAAAACAGATAATGTTAAAGAGAAAATTGACTTCGTAAGAACTGGTGCAAGATTTGATGTTACTCCAAAATCTGGCGTGTATGGAAACTACTCTTATGGTACTTACAAAGATAAAGCTTACAAAGCAACAGCTCATCAATTCATGTTAGGTGCAGACTATAAATTACATAAACAAGTTGTTACCTTTGTTGAAGGTCGTTTAATCAAGAACAAAGACAGTAATAACAAAAAAGTTACTGACCAAGCACTTGGTGTTGGTTTACGTGTATTATGGTAA

>gi|209968847|gb|EU741909.1| Haemophilus parasuis strain No. 4 outer membrane protein P2 (ompP2) gene, complete cds

ATGAAAAAAACACTAGTAGCATTAGCAGTAGCGGCATTTGCAGCATCAGCATCAGCTGTAACAGTTTATGAAAATGAAGGTACAAAAGTTGATTTTGATGGTCAATTGCGTCTTCTTTTAGAAGAACAAGCCACAAAAGAGAAAGGTCAATCTTCAACAGGTGGTCACACTAACTTAAAGAATAATGGTTCTCGTTTCGGTATTTCTATCAAACATAATATCAATGAGAATCTCTACGGTTTTGGTCGTTATGAGACTCGCCTTGGCAGTGGTTCTAAAAATGCTGCAGAATGGGGTGATGTTACAACAGAAGAGGCTTACGTTGGTTTAGGTGGCTATGGTCATGAAATTTCTTTTGGTAAACAAGCTGTAATCGGTGATAGCATTGGTCAAGCTGGTTTTGATAAAGTATACGGTGTTGGTACTGGTGGAATTAAATATTCAGCAAACAACACAAACAAAAAAGGTTTTGATATCCTTACTGCATCTTCTGATTCAGCAATTAACTATACCTATACAGGTATTGAAGGTTTGACGTTAGGTGCTAACTATAATGTTGCAAATGAGCGTGATGATAAGGGAGGCGTAAAAGTAGGTTCTATTAAATCTGGCTTTGGTTTAGGTGCTAAATACACAGCTAAGATTGCGGAAAGTCAATCTGTAACTGTGGCAGCAGGTTATACTCATGATGACTATAAATCTGGAGCTGTTAATAAGAAAGACAAAGATGGTGTATACTTTGGTCTTAAATATGTCAATGCTCCATTTACTGTAGCTGTTGATGGTGGTCATGGTGTTGTAAAAACAGATAATGTTAAAGAGAAAATTGACTTCGTAAGAACTGGCGCAAGATTTGATGTTACTCCAAAATCTGGCGTGTATGGAAACTACTCTTATGGTACTTACAAAGATAAAGCTTACAAAGCAACAGCTCATCAATTCATGTTAGGTGCAGACTATAAATTACATAAACAAGTTGTTACCTTTGTTGAAGGTCGTTTAATCAAGAACAAAGACAGTAATAACAACAAAGTTACTGACCAAGCACTTGGTGTTGGTTTACGTGTATTATGGTAA

>gi|209968845|gb|EU741908.1| Haemophilus parasuis strain 174 outer membrane protein P2 (ompP2) gene, complete cds

ATGAAAAAAACACTAGTAGCGTTAGCAGTAGCGGCATTTGCAGCATCAGCATCAGCTGTAACAGTTTATGAAAATGAAGGTACAAAAGTTGATTTTGATGGTCAATTGCGTCTTCTTTTAGAAGAACAAGCCACAAAAGAGGAAGGTCAATCTTCAACAGGTGGTCACACTAACTTAAAGAATAATGGTTCTCGTTTCGGTATTTCTATCAAACATAATATCAATGAGAATCTCTACGGTTTTGGTCGTTATGAGACTCGCCTTGGCCGTAATTCTAAAAATGATGCAGGATGGGGGGATGTTACAACAGATGAGGCTTACGTTGGTTTAGGTGGCTATGGTCATGAAATTTCTTTTGGTAAACAAGCTGTAATCGGTGATAGCATTGGTCAAGCTGGTTTTGATAAAGTATACGATGTTGGTACTGGTGGAATTAAATATTCAGCAAACAACACAAACAAAAAAGGTTTTGATATCCTTACTGCATCTTCTGATTCAGCAATTAACTATACCTATACAGGCATTGAAGGTTTGACGTTAGGTGCTAACTATAATGTTGCAAATGAGCGTGATAATAAGGGAGGAGTAAAGGTAGATTCTACTAAATCTGGCTTTGGTTTAGGTGCTAAATACACAGCTAAGATTGCGGAAAGTCAATCTGTAACTGTGGCAGCAGGTTATACTCATGATGACTATAAATCTGGAGCTGTTAATAAGAAAGACAAAGATGGTGTATACTTTGGTCTTAAATATGTCAATGCTCCATTTACTGTAGCTGTTGATGGTGGTCATGGTGTTGTAAAAACAGATAATGTTAAAGAGAAAATTGACTTCGTAAGAACTGGTGCAAGATTTGATGTTACTCCAAAATCTGGCGTGTATGGAAACTACTCTTATGGTACTTACAAAGATAAAGCTTACAAAGCAACAGCTCATCAATTCATGTTAGGTGCAGACTATAAATTACATAAACAAGTTGTTACCTTTGTTGAAGGTCGTTTAATCAAGAACAAAGACAGTAATAACAAAAAAGTTACTGACCAAGCACTTGGTGTTGGTTTACGTGTATTATGGTAA

>gi|209968843|gb|EU741907.1| Haemophilus parasuis strain Nagasaki outer membrane protein P2 (ompP2) gene, complete cds

ATGAAAAAAACACTAGTAGCATTAGCAGTAGCGGCATTTGCAGCATCAGCATCAGCTGTAACAGTTTATGAAAATGAAGGTACAAAAGTTGATTTTGATGGTCAATTGCGTCTTCTTTTAGAAGAACAAGCCACAAAAGAGAAAGGTCAATCTTCAACAGGTGGTCACACTAACTTAAAGAATAATGGTTCTCGTTTCGGTATTTCTATCAAACATAATATCAATGAGAATCTCTACGGTTTTGGTCGTTATGAGACTCGCCTTGACAGTAATTCTAAAAATGCTGCAGGATGGGGCGATGTTAAAACAAAATATGCTTACGTCGGTTTAGGTGGCTATGGTCATGAAATTTCTTTTGGTAAACAAGATGTAATCGGTGATAGCATTGGTCAAGCTGGTTTTGATAAAGTATACGGTGTTGGTACTGGTGGAATTAAATATTCAGCAAACAACACAAACAAAAAAGGTTTTGATATCCTTACTTCAGATTCTGATTCAGCAATTAACTATACCTATACAGGCATTGAAGGTTTGACGTTAGGTGCTAACTATAATGTTGCAAATGAGCGTGATAAGAAGACGGGAGAAGTAAATGTAGGTTCTACTAAATCTGGCTTTGGTTTAGGTGCTAAATACACAGCTAAGATTGCGGAAAGTCAATCTGTAACTGTGGCAGCAGGTTATACTCATGATGACTATAAATCTGGATCTGTTAATAAGAAAGACAAAGATGGTGTATACTTTGGTCTTAAATATGTCAACGCTCCATTTACTGTAGCTGTTGATGGTGGTCATGGTGTTGAAAAAACAGGTAATGTTAAAGAGAAAATTGACTTCGTAAGAACTGGCGCAAGATTTGATGTTACTCCAAAATCTGGCGTGTATGGAAACTACTCTTATGGTACTTACAAAGATAAAGCTTACAAAGCAACAGCTCATCAATTCATGTTAGGTGCAGACTATAAATTACATAAACAAGTTGTTACCTTTGTTGAAGGTCGTTTAATCAAGAACAAAGACAGTAATAACAAAAAAGTTACTGACCAAGCACTTGGTGTTGGTTTACGTGTATTATGGTAA

>gi|209968841|gb|EU741906.1| Haemophilus parasuis strain SW114 outer membrane protein P2 (ompP2) gene, complete cds

ATGAAAAAAACACTAGTAGCGTTAGCAGTAGCGACATTTGCAGCATCAGCATCAGCTGTAACAGTTTATGAAAATGAAGGTACAAAAGTTGATTTTGATGGTCAGTTGCGTCTTCTTTTAGAAAAACAAGCCTCAAAAGAGAAAGGTAAATCTTCAACAGATGGTCACACTAACTTAAAGAATAATAGTTCTCGTTTCGGTATTTCTATCAAACATAATATCAATGAGAATCTCTACGGTTTTGGTCGTTATGAGACTCGCCTTGGCAGTGGTTCTAAAAATGCTGCAAAATGGGGTGATGTTACAACAGATGAGGCTTACGTTGGTTTAGGTGGCTATGGTCATGAAATTTCTTTTGGTAAACAAGCTGTAATCGGTGATAGCATTGGTCAAGCTGGTTTTGATAAAGTATACGGTGTTGGTACTGGTGGAATTAAATATACATATGAGGTAGAAGAGTCTATCACTGTGGACAATAAACAGGGTACATTTAAATATTCAGCAGCTCAAGAGGGTTTTGATATCCTTACTCAATCTTCTGATTCAGCAATTAACTATACCTATACAGGTATTGAAGGTTTGACGTTAGGTGCTAACTATAATGTTGCAAATGAGCGTGAGAAGGCAGATGTAAAAGTAGATTCTATTAAATCTGGCTTTGGTTTAGGTGCTAAATACACAGCTAAGATTGCAGAAAGTCAATCTGTAACTGTGGCAGCAGGTTATACTCATGATGACTACAAATCTGGATCTGTTAAACTAAAAGGTAAATTTGTTGAAGCAGGTGGTAAATCTACAGACCATACCTATACAAAAAAACCATTTAATAAGAAAGACAAAGATGGTGTGTACTTTGGTCTTAAATATGTCAACGCTCCATTTACTGTAGCTGTTGATGGTGGTCATGGTGTTGTAAAAACAGATGATGTTAAAGAGAAAATTAACTTCGTAAGAACTGGCGCAAGATTTGATGTTACTCCAAAATCTGGCGTGTATGGAAACTACTCTTATGGTACTTATAAAGTTGAAGATTTCAAAGTAACTGCTCATCAATTCATGTTAGGTGCAGACTATAAATTACATAAACAAGTTGTTACCTTTGTTGAAGGTCGTTTAATCAAGAACAAAGACAGTGATAACAACAAAGTTACTGACAAAGCACTTGGTGTTGGTTTACGTGTATTATGGTAA

>gi|209968839|gb|EU741905.1| Haemophilus parasuis strain SW140 outer membrane protein P2 (ompP2) gene, complete cds

ATGAAAAAAACACTAGTAGCGTTAGCAGTAGCGGCATTTGCAGCATCAGCATCAGCTGTAACAGTTTATGAAAATGAAGGTACAAAAGTTGATTTTGATGGTCAATTGCGTCTTCTTTTAGAAAAACAAGCCTCAAAAGTGAAAGGTCAATCTTCAACAAGTGGTCACACTGACTTAAAGAATAATGGTTCTCGTTTCGGTATTTCTATCAAACATAATATCAATGAGAATCTCTACGGTTTTGGTCGTTATGAGACTCGCCTTGACAGTAATTCTAAAAATGCTGCAGGATGGGGCGATGTTAAAACAAAATATGCTTACGTCGGTTTAGGTGGCTATGGTCATGAAATTTCTTTTGGTAAACAAGTTGTAATCGGTGATAGCATTGGTCAAGCTGGTTTTGATAAAGTATACGATGTTGGTAGTACTGGAATTAGAATTAAATATCCAGCAAACTCAGCAGACAAAAAAGGTTTTGATATCCTTACTTCAGATTCTGATTCAGCAATTAACTATACCTATACAGGCATTGAAGGTTTGACGTTAGGTGCTAACTATAATGTTGCAAATGAGCGTGATAAGAAGACGGGAGAAGTAAAAGTAGATTCTGCTAAATCTGGCTTTGGTTTAGGTGCTAAATACACAGCTAAGATTGCGGAAAGTCAATCTGTAACTGTGGCAGCAGGTTATACTCATGATGACTATAAATCTGGAGCTGTTAATAAGAAAGACAAAGATGGTGTATACTTTGGTCTTAAATATGTCAATGCTCCATTTACTGTAGCTGTTGATGGTGGTCATGGTGTTGTAAAAACAGATAATGTTAAAGAGAAAATTGACTTCGTAAGAACTGGTGCAAGATTTGATGTTACTCCAAAATCTGGCGTGTATGGAAACTACTCTTATGGTACTTACAAAGATAAAGCTTACAAAGCAACAGCTCATCAATTCATGTTAGGTGCAGACTATAAATTACATAAACAAGTTGTTACCTTTGTTGAAGGTCGTTTAATCAAGAACAAAGACAGTAATAACAAAAAAGTTACTGACAAAGCACTTGGTGTTGGTTTACGTGTATTATGGTAA

>gi|209968837|gb|EU741904.1| Haemophilus parasuis strain D74 outer membrane protein P2 (ompP2) gene, complete cds

ATGAAAAAAACACTAGTAGCGTTAGCAGTAGCGACATTTGCAGCATCAGCATCAGCTGTAACAGTTTATGAAAATGAAGGTACAAAAGTTGATTTTGATGGTCAGTTGCGTCTTCTTTTAGAAAAACAAGCCTCAAAAGAGAAAGGTAAATCTTCAACAGATGGTCACACTAACTTAAAGAATAATAGTTCTCGTTTCGGTATTTCTATCAAACATAATATCAATGAGAATCTCTACGGTTTTGGTCGTTATGAGACTCGCCTTGGCAGTGGTTCTAAAAATGCTGCAAAATGGGGTGATGTTACAACAGATGAGGCTTACGTTGGTTTAGGTGGCTATGGTCATGAAATTTCTTTTGGTAAACAAGCTGTAATCGGTGATAGCATTGGCCAAGCTGGTTTTGATAAAGTATACGGTGTTGGTACTGGTGGAATTAAATATACATATGAGGTAGAAGAGTCTATCACTGTGGACAATAAACAGGGTACATTTAAATATTCAGCACCTCAAGAAGGTTTTGATATCCTTACTCAATCTTCTGATTCAGCAATTAACTATACCTATACAGGTATTGAAGGTTTGACGTTAGGTGCTAACTATAATGTTGCAAATGAGCGTGAGGAGGCAGATGTAAAAGTAGATTCTATTAAATCTGGCTTTGGTTTAGGTGCTAAATACACAGCTAAGATTGCAGAAAGTCAATCTGTAACTGTGGCAGCAGGTTATACTCATGATGACTACAAATCTGGATCTGTTAAACTAAAAGGTAAATTTGTTCAAGCAAATGGTACATCTACAGACCATATCCATACAGAAAAACCATTTAATAAGAAAGACAAAGATGGTGTGTACTTTGGTCTTAAATATGTCAACGCTCCATTTACTGTAGCTGTTGATGGTGGTCATGGTGTCGTAAAAAAAGATGATGTTAAAGAGAAAATTAACTTCGTAAGAACTGGCGCAAGATTTGATGTTACTCCAAAATCTGGCGTGTATGGAAACTACTCTTATGGTACTTATAAAAAAGTTGAAGATTTCAAAGTAACTGCTCATCAATTCATGTTAGGTGCAGACTATAAATTACATAAACAAGTTGTTACCTTTGTTGAAGGTCGTTTAATCAAGAACAAAGACAGTGATAACAACAAAGTTACTGACAAAGCACTTGGTGTTGGTTTACGTGTATTATGGTAA
